# In silico identification of potential calcium dynamics and sarcomere targets for recovering left ventricular function in rat heart failure with preserved ejection fraction

**DOI:** 10.1371/journal.pcbi.1009646

**Published:** 2021-12-06

**Authors:** Stefano Longobardi, Anna Sher, Steven A. Niederer

**Affiliations:** 1 Cardiac Electromechanics Research Group, School of Biomedical Engineering and Imaging Sciences, King’s College London, London, UK; 2 Pfizer Worldwide Research, Development and Medical, Cambridge, Massachusetts, United States of America; University of Virginia, UNITED STATES

## Abstract

Heart failure with preserved ejection fraction (HFpEF) is a complex disease associated with multiple co-morbidities, where impaired cardiac mechanics are often the end effect. At the cellular level, cardiac mechanics can be pharmacologically manipulated by altering calcium signalling and the sarcomere. However, the link between cellular level modulations and whole organ pump function is incompletely understood. Our goal is to develop and use a multi-scale computational cardiac mechanics model of the obese ZSF1 HFpEF rat to identify important biomechanical mechanisms that underpin impaired cardiac function and to predict how whole-heart mechanical function can be recovered through altering cellular calcium dynamics and/or cellular contraction. The rat heart was modelled using a 3D biventricular biomechanics model. Biomechanics were described by 16 parameters, corresponding to intracellular calcium transient, sarcomere dynamics, cardiac tissue and hemodynamics properties. The model simulated left ventricular (LV) pressure-volume loops that were described by 14 scalar features. We trained a Gaussian process emulator to map the 16 input parameters to each of the 14 outputs. A global sensitivity analysis was performed, and identified calcium dynamics and thin and thick filament kinetics as key determinants of the organ scale pump function. We employed Bayesian history matching to build a model of the ZSF1 rat heart. Next, we recovered the LV function, described by ejection fraction, peak pressure, maximum rate of pressure rise and isovolumetric relaxation time constant. We found that by manipulating calcium, thin and thick filament properties we can recover 34%, 28% and 24% of the LV function in the ZSF1 rat heart, respectively, and 39% if we manipulate all of them together. We demonstrated how a combination of biophysically based models and their derived emulators can be used to identify potential pharmacological targets. We predicted that cardiac function can be best recovered in ZSF1 rats by desensitising the myofilament and reducing the affinity to intracellular calcium concentration and overall prolonging the sarcomere staying in the active force generating state.

## Author summary

We developed a computational model of the ZSF1 rat model of heart failure with preserved ejection fraction. We validated that the model can link simulated pharmacological interventions from cellular to whole heart pump function. Our computational model identified calcium dynamics as the main determinant of left ventricular contractile behaviour. We demonstrated that the highest degree of LV function recovery could be achieved when calcium dynamics is manipulated in conjunction with both thin and thick filament kinetics.

## 1 Introduction

Heart failure (HF) is a progressive and prevalent disease. Approximately 50% of patients have heart failure with preserved ejection fraction (HFpEF), characterised by impaired myocardial relaxation and often secondary to hypertension and obesity. There are limited evidence-based pharmacotherapies for HFpEF and thus HFpEF represents an unmet clinical need. Patients currently receive either angiotensin-converting enzyme inhibitors/aldosterone receptor blockers, calcium channel blockers or beta-blockers, but the mortality and the morbidity associated with the disease have so far remained high [1].

Animal models constitute a valuable research tools to investigate HFpEF, as comorbidities and other confounding factors can be more precisely controlled than in the clinical setting. However, there are no perfect animal models for HFpEF, and this is in part because it is difficult to fulfil all the features observed in human disease at the same time in animals. The currently available animal models of HFpEF have attempted to reproduce the dominant factors typically documented to cause diastolic dysfunction and HFpEF. They fall across the following macro-categories: aortic banding and systemic hypertension, diabetes mellitus and obesity, cardiometabolic syndrome and ageing. All of these animal models have been successfully established in rodents [2]. Regardless of the animal model used in the process of drug discovery and development at preclinical stages, identifying pharmacological interventions that recover physiological function in the HFpEF diseased animal still remains a challenge.

In this study we aim to predict changes in myocyte function that recovers whole heart function. First, we propose a multi-scale mathematical model that maps ion channel and sarcomere function through to whole organ pump function in a HFpEF rat heart. Specifically, we want to build an *in silico* representation of the *20-week old obese ZSF1 rat*, a recently proposed HFpEF animal model. This model can then be used to identify cellular function that can be manipulated to recovered whole heart function. We propose to use this animal model to provide indications of pharmacological targets by simulating and testing their different mechanisms of action. The 20-week old obese ZSF1 rat presents many features of a cardiometabolic syndrome such as hypertension, obesity, type 2 mellitus, insulin resistance and HF, developing a diastolic dysfunction in parallel with left ventricular (LV) hypertrophy and left atrial dilation. As this animal model also presents exercise intolerance, an important feature diagnosed in humans, it currently constitutes a well-established [2] animal model of HFpEF. From now on, we will refer to the “20-weeks old obese ZSF1 rat” as the “ZSF1 rat” for brevity.

## 2 Materials and methods

### 2.1 Rat heart contraction model

We modelled the healthy rat heart using a 3D biventricular contraction model previously [3] fitted to anatomic, structural, and hemodynamic and volumetric functional data from sham-operated controls [4]. This rat model will be referred to as “SHAM” throughout the entire work. At the cellular level, ion fluxes and calcium dynamics were simulated using the Gattoni et al. [5] model of rat left ventricular myocyte electrophysiology at 37° and 6 HZ pacing frequency. Active tension generation was described using the Land et al. [6] model of sarcomere contraction, comprising thin and thick filament dynamics, and accounting for sarcomere-length and -velocity dependencies. Left and right ventricular (RV) anatomy was represented by a cubic Hermite finite element mesh [7] fitted to manually segmented MRI images related to a time point of the cardiac cycle which was half-way through diastole to approximate the stress-free configuration. Rule-based fibres [8] were included, with a transmural variation of −60° to 80° from epicardium to endocardium. Passive material properties were modelled using the transversly isotropic cardiac strain energy function proposed by Guccione [9]. This was further combined with a Lagrange multiplier scheme to enforce incompressibility, coupled with a penalty term to improve stability of mechanics simulations [10, 11]. The calcium transient was assumed not to vary spatially and it homogeneously activated contraction throughout the ventricular walls. Spatial boundary conditions were applied by constraining all the ventricle basal plane mesh nodes along the apex-base axis, and by allowing no movement along any direction for one mesh node on the interior LV wall to prevent free rotation and translation of the mesh during the solution process without limiting the deformation [6]. The hemodynamics (blood flow and pressures) at the computational domain boundaries was controlled by coupling the heart with the rest of the body circulatory system via a three-element Windkessel model [12].

The whole-organ full cardiac cycle was simulated using the protocol described in [6, 10, 11]. Briefly, dynamic changes in the LV and RV cavities’ boundary conditions were cyclically applied. During diastole, a fixed atrial preload pressure and filling resistance was applied. At activation, the cavity boundary conditions were switched to an isovolumetric constraint in both chambers. When each chamber reached pre-set aortic and pulmonary artery pressures, a three-element Windkessel model boundary condition was applied to each chamber to represent the aortic and pulmonary artery afterload. Once each chamber stopped ejecting, an isovolumetric boundary condition was applied to represent isovolumetric contraction. Once the cavity pressure fell below its respective atrial pressure, the heart returned to the diastolic cavity boundary conditions to complete the pressure volume (PV) loop. The RV boundary condition pressures (atrial and pulmonary artery) were set to be 1/3 of the equivalent LV values. The RV Windkessel parameters were scaled to be equal to *R*/3, *Z*/3, 3*C* from the reference LV Windkessel parameter set (*R*, *Z*, *C*).

The presented multi-scale rat heart contraction model is regulated by 71, 17, 18 parameters for respectively the ionic, cell contraction, tissue + boundary components. We selected specific parameters as representative regulators of each of these sub-models, for a total of 16 parameters. The calcium transient represented the end output of the cellular electrophysiology model used for the sarcomere contraction model, and was simulated only once at this stage. This is because the first 4 parameters we selected for the multi-scale map input encoded the whole shape of the calcium transient and could independently scale its main properties (diastolic concentration, amplitude, time to peak and recovery time, see S1 Text for more details). Of the other selected parameters, 8 described the sarcomere dynamics (4 parameters were thin filament-related and 4 were thick filament-related), and 4 parameters described boundary conditions and tissue properties. The 16 parameters considered are defined in Table 1.

**Table 1:** Model input parameters.

Parameter	Units	Definition
DCA	μm	diastolic Ca^2+^ concentration
AMPL	μm	Ca^2+^ concentration signal amplitude
TP	ms	time to peak Ca^2+^ concentration
RT50	ms	time to half-maximal relaxation from peak Ca^2+^ concentration
Ca_50_	μm	Ca^2+^ thin filament sensitivity
*β*_1_	−	phenomenological tension length-dependence scaling factor
*k*_off_	ms^-1^	unbinding rate of Ca^2+^ from TnC
*n*_trpn_	−	Ca^2+^-TnC binding degree of cooperativity
*k*_xb_	ms^-1^	cross-bridges cycling rate
*n*_xb_	−	cross-bridge formation degree of cooperativity
TRPN_50_	−	fraction of Ca^2+^-TnC bounds for half-maximal cross-bridges activation
*T*_ref_	kPa	maximal reference tension
*p*	kPa	end-diastolic pressure
*p*_ao_	kPa	aortic systolic pressure
*Z*	mmHg s mL^-1^	aortic characteristic impedance
*C*_1_	kPa	tissue stiffness

The rat heart model output is given as the LV pressure and volume transients, and the related PV loop. To quantitatively characterise the LV activity, we extracted from these two curves 14 scalar features of interest which are commonly used to characterise LV systolic and diastolic functions (Table 2). The process of features extraction is illustrated in S2 Text.

**Table 2:** Model output LV features.

Label	Units	Definition
EDV	μm	end-diastolic volume
ESV	μm	end-systolic volume
SV	μm	stroke volume
EF	%	ejection fraction
IVCT	ms	isovolumetric contraction time
ET	ms	systolic ejection time
IVRT	ms	isovolumetric relaxation time
Tdiast	ms	diastolic filling time
PeakP	kPa	peak systolic pressure
Tpeak	ms	time to peak systolic pressure
ESP	kPa	end-systolic pressure
maxdP	kPa ms^-1^	maximum pressure rise rate
mindP	kPa ms^-1^	maximum pressure decay rate
Tau	ms	isovolumetric pressure relaxation time constant

For a given heart mesh describing the cardiac anatomy and fibre orientation, we can define a multi-scale, non-linear function that maps every set of 16 input parameters **x** to a set of 14 output LV features (*y*_1_, …, *y*_14_):

$$\begin{array}{cc} f_{simul}:R^{16} & \to \underset{14\;\text{times}}{\underset{︸}{R\times \ldots \times R}}\\ x & \mapsto (y_{1},\;\ldots ,\;y_{14}) \end{array}$$ (1)

**Fig 1:**
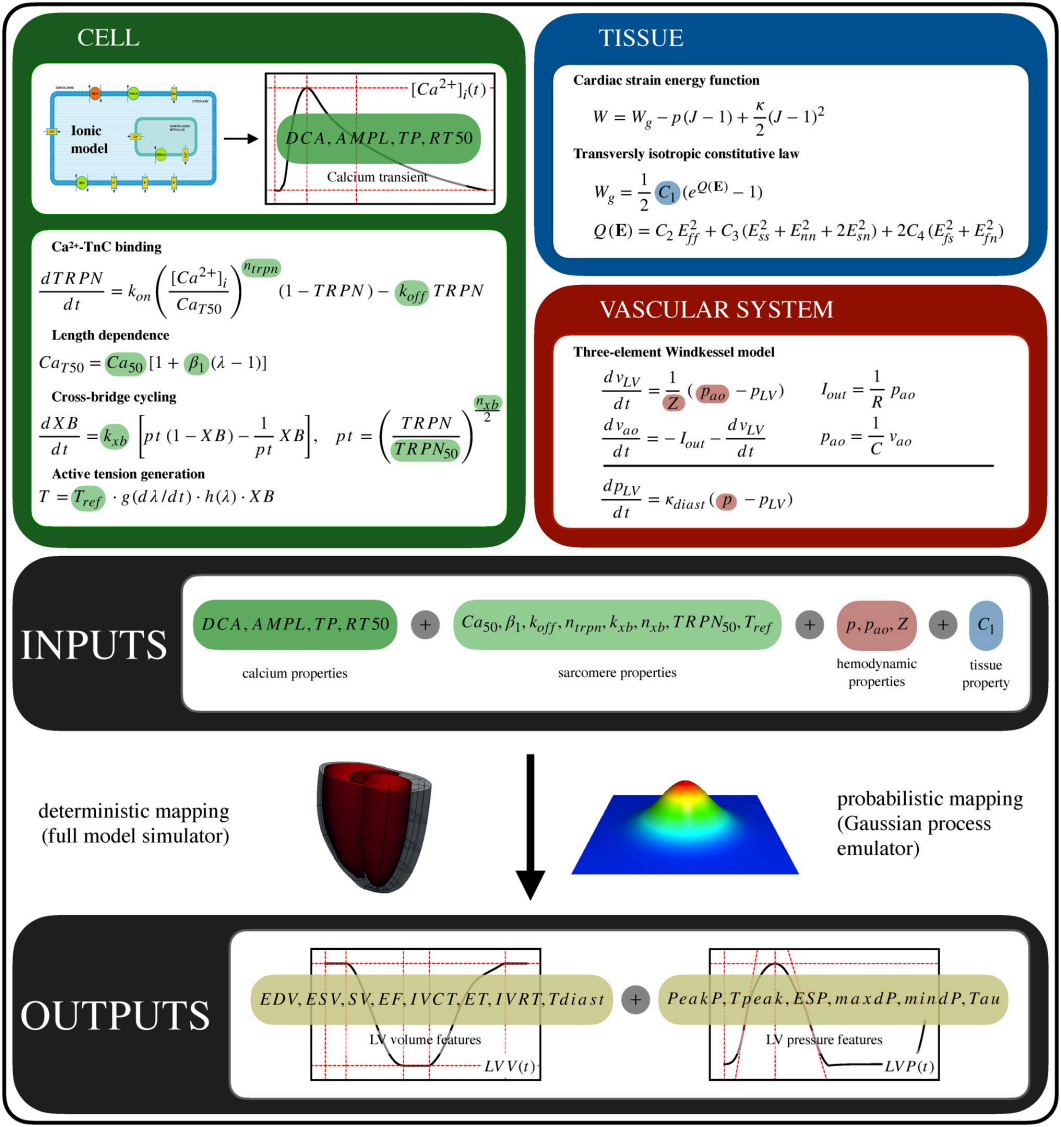
3D biventricular rat heart contraction model multi-scale map. Chosen 16 input parameters are calcium transient and sarcomere properties (green), hemodynamics properties (red) and tissue properties (blue). The output features of interest are 14 indexes (yellow) characterising the LV function and are extracted from the LV pressure and volume curves. The input parameters (Table 1) can be quantitatively be mapped to the output features (Table 2) either by running the full model or by making predictions using trained GPEs.

Eq (1) effectively constitutes a quantitative link between cellular, tissue and hemodynamic properties to whole-organ function. Parameters can be mapped to features by running the full forward model (*simulator*). However, this is computationally expensive (∼4–10 CPU hours per evaluation). To reduce computational costs we trained a low cost Gaussian process emulator (GPE) to be a surrogate for the full model (Section 2.2. Fig 1 provides a schematic of the multi-scale mapping: after training the emulator, we will be able to map input parameters to output LV features both in a deterministic (using the simulator) and in a probabilistic (using the emulator) way.

### 2.2 Surrogate model

GP emulation was employed to replace the computationally expensive map from model input parameters to model output LV features. We followed the same emulation framework as in [3]. Briefly, we trained the emulators to simulations with parameter values sampled across a 16-dimensional parameter space, defined as the hypercube obtained by the Cartesian product of individual, 1-dimensional parameter intervals. Each parameter interval was constructed with lower and upper bounds given as percentages of the SHAM rat heart model reference parameter values, based on a literature search and preliminary sensitivity analysis studies (see S3 Text for more details). For the sarcomere, boundary conditions and material properties we varied parameters in the range [50%, 150%], except for *β*_1_ which we varied in the range [10%, 200%]. To cover both healthy and pathological calcium transient shapes, DCA, AMPL and TP parameters were varied in the range [10%, 200%], while for RT50 a smaller range was used ([10%, 110%]). This was done to limit the generation of implausible calcium transients (where the sum of the time to peak and the relaxation time exceed the cycle length) when randomly scaling the reference calcium transient (see S1 Text for more details).

14, 848 points were sampled from a Latin hypercube design over the input parameter space. The forward model was run at these points and the successfully completed simulations were collected to form the training dataset. The final dataset consisted of 1, 299 data points, corresponding to 8.7% of the total simulations attempted. A visual and quantitative inspection of the obtained training dataset input parameter space suggested (see S3 Text for more details) that this was still able to cover over 80% of the initial sampling space. The low number of successful runs relates to a combination of failure of the mechanics simulations to converge or complete a full cardiac cycle, which may happen for example if the contraction is insufficient to reach the aortic pressure.

GPEs *f*(**x**) were defined as the sum of a deterministic mean function *h*(**x**) and a stochastic process *g*(**x**) [13]:

$$\begin{array}{c} f(x)=h(x)+g(x) \end{array}$$ (2)

The mean function was a linear regression model with first-order degree polynomials:

$$\begin{array}{c} h\left(x\right)≔\beta_{0}+\beta_{1}x_{1}+\ldots +\beta_{16}x_{16} \end{array}$$ (3)

where $\beta_{i}\in R\;\;\text{for}\;\;i=0,\ldots ,16$ are the weights, while the stochastic process was a centred (zero-mean) Gaussian process with the stationary squared exponential kernel as covariance function:

$$\begin{array}{cc} & g\left(x\right)∼GP(0,\;k_{\text{SE}}\left(d\left(x,\;x^{\prime }\right)\right)) \end{array}$$ (4)

$$\begin{array}{cc} & k_{\text{SE}}\left(d\left(x,\;x^{\prime }\right)\right)≔\sigma_{f}^{2}\;e^{-\frac{1}{2}\;d\left(x,\;x^{\prime }\right)} \end{array}$$ (5)

$$\begin{array}{cc} & d\left(x,\;x^{\prime }\right)≔{\left(x-x^{\prime }\right)}^{T}\;\Lambda \;\left(x-x^{\prime }\right) \end{array}$$ (6)

where $\sigma_{f}^{2}\in R^{+}$ is the signal variance and $\Lambda ≔\text{diag}(\ell_{1}^{2},\ldots ,\ell_{16}^{2})$, $\ell_{i}\in R^{+}$ for *i* = 1, …, 16 are the characteristic length-scales of the process. The model likelihood was taken to be Gaussian, i.e. the learning sample observations (*y*) were modelled to be affected by an additive, independent and identically distributed noise:

$$\begin{array}{c} y=f\left(x\right)+\varepsilon ,\;\varepsilon ∼N\left(0,\;\sigma_{n}^{2}\right) \end{array}$$ (7)

where $\sigma_{n}^{2}\in R^{+}$ is the noise variance. All the GPE’s hyperparameters were jointly optimised during training by maximisation of the model log-marginal likelihood using GPErks emulation tool [14] based on GPyTorch Python library [15].

Univariate GPEs were trained to predict each output feature using a 5-fold cross-validation process, for a total of 14 trained GPEs. To evaluate each emulator’s accuracy, the predicted posterior mean emulator output $y_{i}^{\text{mean}}$ was compared with the true output value $y_{i}^{\text{true}}$ for each respective point **x**_*i*_ in a held-out testing dataset of size *n* × 16. We used the coefficient of determination (or *R*^2^-score) to measure how well the regression predictions approximate the real data points. This is defined as:

$$\begin{array}{c} R^{2}≔1-\frac{\sum_{i=1}^{n}{\left(y_{i}^{\text{true}}-y_{i}^{\text{mean}}\right)}^{2}}{\sum_{i=1}^{n}{\left(y_{i}^{\text{true}}-\bar{y}\right)}^{2}},\;\text{with}\;\bar{y}≔\frac{1}{n}\sum_{i=1}^{n}y_{i}^{\text{true}} \end{array}$$ (8)

We additionally used the predicted posterior variance emulator outputs $y_{i}^{\text{var}}$, for *i* = 1, …, *n* to calculate the percentage of points which had an independent standard error (ISE) smaller than 2. This quantity, which we call ISE_2_, is a measure of how well the emulator uncertainty is accounting for the mean predictions’ departure from the observed data, and is defined as:

$$\begin{array}{c} {\text{ISE}}_{2}≔100\cdot \sum_{i=1}^{n}(\frac{|y_{i}^{\text{true}}-y_{i}^{\text{mean}}|}{\sqrt{y_{i}^{\text{var}}}}<2)/n \end{array}$$ (9)

The Boolean result inside the parentheses is encoded with either 0 (false) or 1 (true). The GPEs’ accuracy was therefore given as the *R*^2^-score and ISE_2_ obtained by averaging the scores calculated when testing the emulators on the respective left-out parts of each dataset splitting during cross-validation. These are summarised in S3 Text, where an example of GPEs doing inference on a testing set is also provided. As a result of the cross-validation procedure, the emulators were tested for accuracy against different regions of the input parameter space, eventually covering all of it.

### 2.3 Model fitting

A Bayesian history matching (HM) technique was used to re-fit model parameters as done previously [3], to create a mathematical model of the obese ZSF1 rat (Section 3.3) and to virtually recover it towards an healthy condition (Section 3.4).

HM is an iterative algorithm that at each iteration (also called *wave*) identifies from the current plausible parameter space (at the first wave this coincides with the full space) the region where parameter sets are more plausible to yield a model output that matches a set of experimentally measured quantities within experimental uncertainty. Every point in the current plausible space is tested against an *implausibility criterion* to determine if it has to be kept for the next wave (*non-implausible*) or discarded (*implausible*). Let *m* be the number of output features to match. Then, for each test point **x**, the trained univariate GPEs $f_{emul}^{i}$ for *i* = 1, …, *m* are used to calculate the following *implausibility measure*:

$$\begin{array}{c} \underset{i=1,\ldots ,m}{\text{max}}\frac{\left|E\left[f_{emul}^{i}\left(x\right)\right]-\mu_{i}\right|}{\sqrt{V\text{ar}\left[f_{emul}^{i}\left(x\right)\right]+\sigma_{i}^{2}}} \end{array}$$ (10)

where $\mu_{i}\pm \sigma_{i}\in R$ represents the experimental variability observed for feature *i*. The implausibility criterion simply consists in comparing the implausibility measure (Eq (10)) with a cutoff value which is normally taken to be equal to 3. An implausibility measure below the cutoff will deem the point non-implausible or implausible if otherwise. At the next wave, the GPEs training dataset is augmented with parameter points from the plausible region of the current wave, so that the re-trained GPEs become more accurate in the plausible region. Points are tested again against the implausibility criterion to find plausible regions and the iterations will continue until reaching convergence of the plausible space.

### 2.4 Global sensitivity analysis

In order to understand the input parameters impact on the output features total variance we performed a global sensitivity analysis. Model outputs sensitivity to parameters was characterised by Sobol’ first-order and total effects [16]. These indexes were estimated using the Saltelli method [17] with SaLib Python library [18]. GPErks tool [14] was used to incorporate full GPE’s posterior distribution samples to account for emulators uncertainty in Sobol’ indexes estimates. Parameters whose resulting indexes were below the threshold 0.01 were determined to have negligible effect.

### 2.5 Model validation

**Fig 2:**
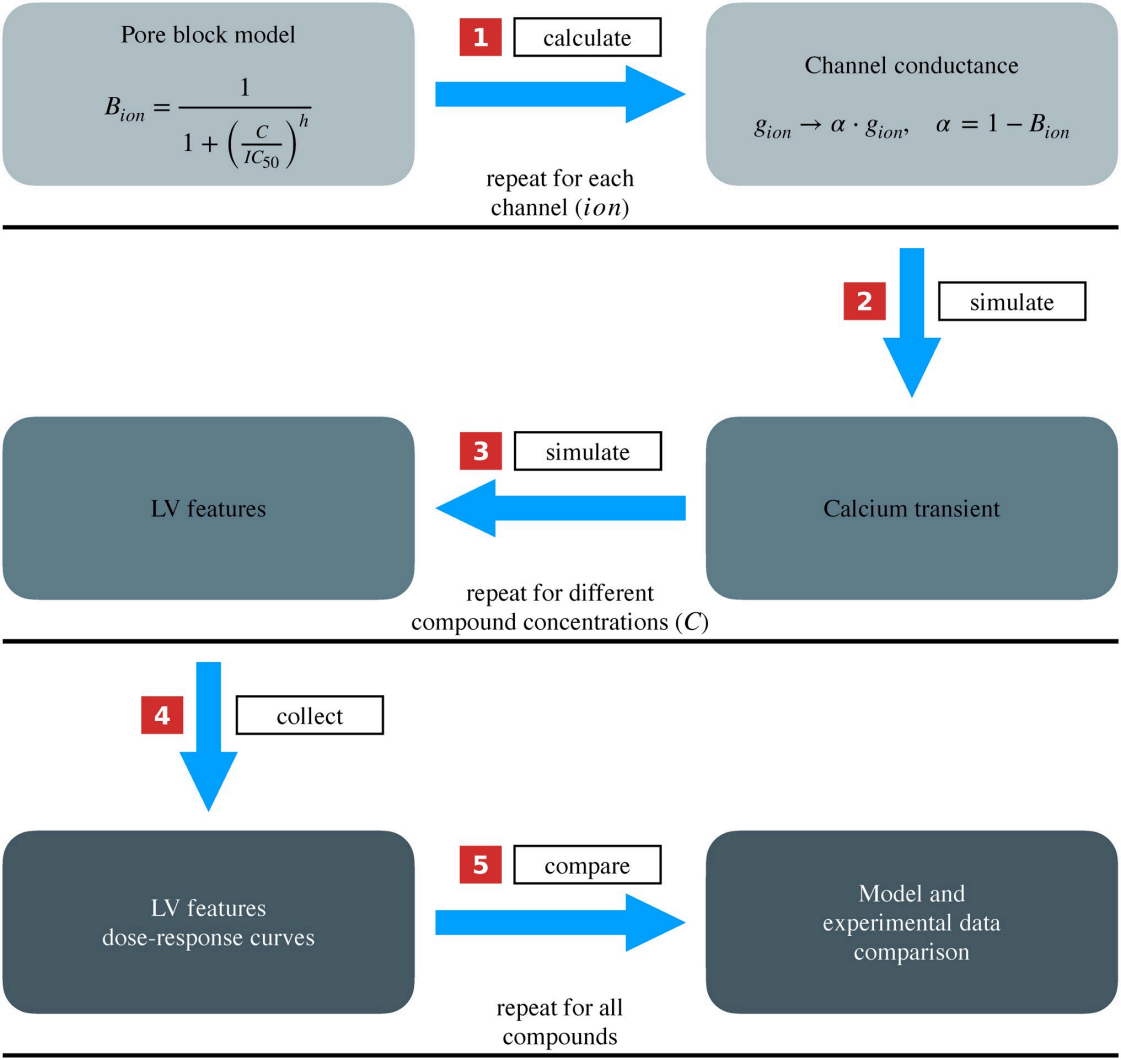
Model validation workflow. (1) The pore block model is used to calculate the fraction of blocked ion channel at a given compound concentration for each ion channel. These channels’ conductances are scaled to reflect the effect of the compound. (2) The rat myocyte electrophysiological model is run to generate perturbed calcium transients for different compound concentrations. (3) The calcium transients are used as an input for the 3D biventricular rat heart contraction model, and perturbed LV features’ values are obtained. For each LV feature, the number of perturbed values was equal to the number of input calcium curves, which corresponds to the number of tested compound concentrations. (4) Each LV feature values are plotted against the tested compound concentrations to obtain dose-response curves. (5) The qualitative trend of the LV features after *in silico* pharmacological modulation is compared with literature experimentally measured effects of the same compound on the same LV features for each compound under study.

To test if the rat heart contraction model and its probabilistic surrogate can predict how changes in calcium transients impact the whole heart function, we validated the computational framework by comparing qualitative measurements and predictions of changes in cardiac mechanics in the presence of pharmacological compounds that manipulate the calcium transient. This provides a multi-scale test on the ability of the model to map from changes in ion channels conductances to changes in calcium transient and the resulting changes in whole heart function. The validation workflow is summarised in Fig 2.

Briefly, we selected 8 compounds, which were well characterised for multiple ion channels and for which we could find whole organ measurements from literature, from the comprehensive in vitro proarrhythmia assay (CiPA) [19] official list, namely bepridil, chlorpromazine, diltiazem, mexiletine, nifedipine, ranolazine, sotalol and verapamil, and we described their action at the cell level using a 4-channel description, namely I_Na_, I_to_, I_K1_ and I_CaL_. I_Kr_ channel was not included as it has a small amplitude in rats myocytes [20] and so was not included in the employed rat cell model [5].

The affinity of each compound for each channel was taken from CiPA project datasets [21, 22] (summarised in S4 Text) and is described by the Hill coefficient *h* and the half-maximal inhibitory concentration IC_50_ values of a Hill-type relationship which gives the fraction of blocked current *B* as a function of the compound concentration *C*, also known as pore block model:

$$\begin{array}{c} B=\frac{1}{1+{\left(\frac{C}{{\text{IC}}_{50}}\right)}^{h}} \end{array}$$ (11)

For each given compound, we calculated *B* for each channel when *C* was set to equally-spaced values in a log-molar space. By subtracting the obtained *B* values from 1, we obtained a matrix of scaling coefficients for the channels’ conductances, representing the fractions of active channels in the presence of the compounds at different concentrations. We tested 13 equally-spaced compound concentrations (−log m) in the range [4, 10] (extremes included), which corresponded to compound concentrations between 10^−10^ m and 10^−4^ m. We then run the Gattoni et al. [5] model by scaling the ion channels conductances to simulate the action of different concentrations of each compound at the cell level and collected the resulting calcium transients (last beat curves of limit cycle, 5000 beats simulations). An example of calcium transients obtained after simulating the effect of verapamil is provided in S4 Text. The used approach simulates the pharmacologically induced changes in calcium transients, thereby overcoming the issue when no directly recorded calcium transients are available. As a result, we created a fully simulated map from cellular pharmacological modulation to whole organ mechanical function.

We used the full multi-scale model (Section 2.1) to simulate the LV features values using as an input the obtained calcium transients and the remaining parameters fixed to the SHAM rat model reference values. This allowed us to obtain the LV features’ change from baseline values in a dose-dependent manner. PeakP, maxdP and mindP features’ simulated responses to different doses of all the tested compounds are reported in S4 Text. These features’ qualitative responses after *in silico* pharmacological modulation were compared to qualitative changes in the same LV features observed after compounds’ administration in literature experimental studies performed on either conscious, or Langendorff-perfused or working healthy rat heart preparations. These experimental changes were either recorded after a single dose in the pre-ischemic phase of an ischemia-reperfusion experiment, or in a dose-dependent manner, and are summarised in S4 Text.

## 3 Results

We can summarise the results as follows. In Section 3.1, we use the trained framework for evaluating the whole multi-scale model output global sensitivities to model parameters. The full model and the surrogate model are validated in Section 3.2 against known pharmacological effects on whole-organ function from literature experimental studies. In Section 3.3, we create a virtual representation of the ZSF1 rat. In Section 3.4, we employ the validated framework to provide indication of both calcium dynamics and sarcomere potential pharmacological targets for *in silico* recovery of the diseased HFpEF rat back to the healthy state.

### 3.1 Model output explained variance

**Fig 3:**
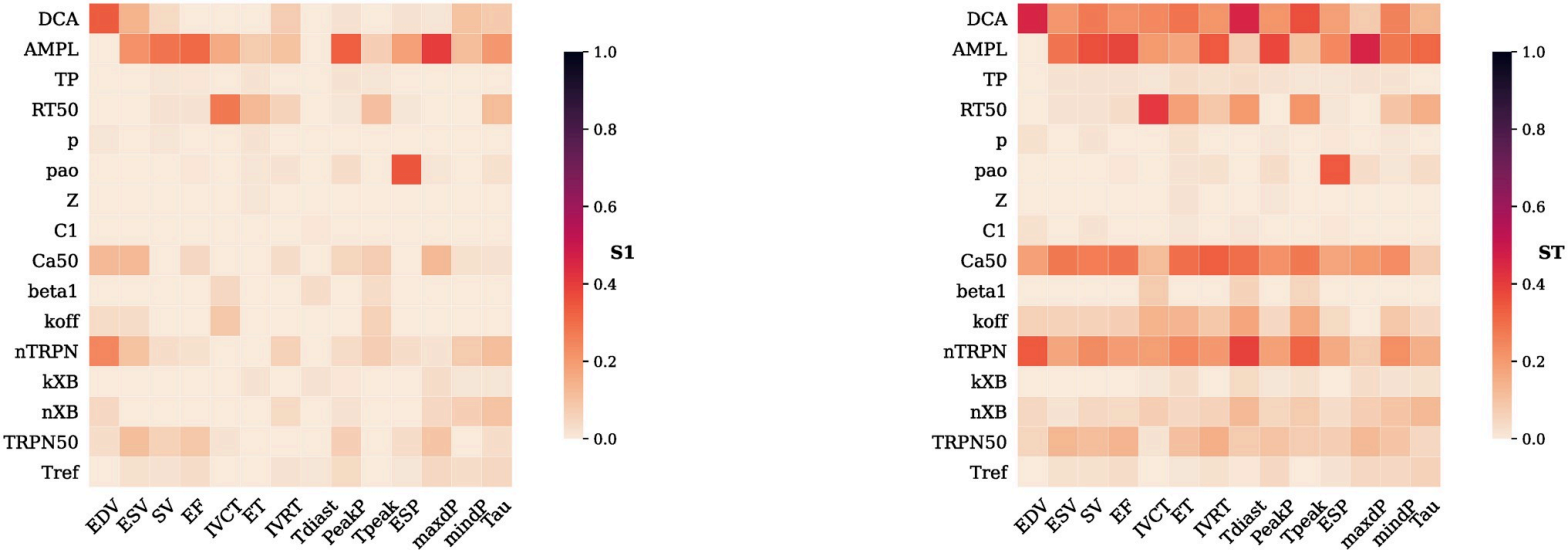
Sobol’ first-order (S1) and total effects (ST). The contribution of each parameter (Table 1) by itself (S1) or jointly with the other parameters (ST) into explaining the total variance of each LV feature (Table 2) is expressed as Sobol’ indices ranging from 0 (no effect) to 1 (maximal effect).

In Fig 3, the calculated Sobol’ first-order and total effects are reported for all the parameters and LV features.

Compounds may target calcium dynamics, thin filament kinetics, thick filament kinetics or tissue/organ scale properties (boundary conditions and stiffness). To determine which of these sets has the greatest impact overall on whole heart cardiac mechanics, we ranked the parameters according to their total effects from the one that affected the highest number (and by the highest amount) to the one that affected the lowest number (and by the lowest amount) of LV features. The obtained ranking is presented in Table 3. We divided the 16 parameters in 4 sub-categories according to which specific part of the multi-scale model they regulated, with 4 parameters in each category. By summation of the parameters’ individual ranks within each category we were able to classify the groups according to how important they are in explaining the variance across output variables: the lower the sum, the higher the importance. We found that the calcium transient is the most important input of the multi-scale model, immediately followed by the thin filament and the thick filament. The boundary conditions were found not to play an important role in this model (ranked fourth). Altering preload and afterload are predicted to have a secondary impact on the overall cardiac function, with cellular properties being the dominant regulators of cardiac function.

**Table 3:** Parameters ranking according to their influence on the model output total variance. A rank is assigned to each parameter according to how much it impacts the model output total variance. Parameter groups are assigned a score given by the sum of the ranks of their member parameters. This score reflects the importance of the group as an input for the multi-scale model.

Group	Parameter	Rank	Group score
Calcium transient (Ca)	DCA	1	22
	AMPL	3	
	TP	10	
	RT50	8	
Thin filament (TNF)	Ca_50_	2	26
	*β*_1_	14	
	*k*_off_	6	
	*n*_trpn_	4	
Thick filament (TKF)	*k*_xb_	12	33
	*n*_xb_	7	
	TRPN_50_	5	
	*T*_ref_	9	
Boundary conditions (BC)	*p*	13	55
	*p*_ao_	11	
	*Z*	16	
	*C*_1_	15	

### 3.2 Model agreement with experiments

**Fig 4:**
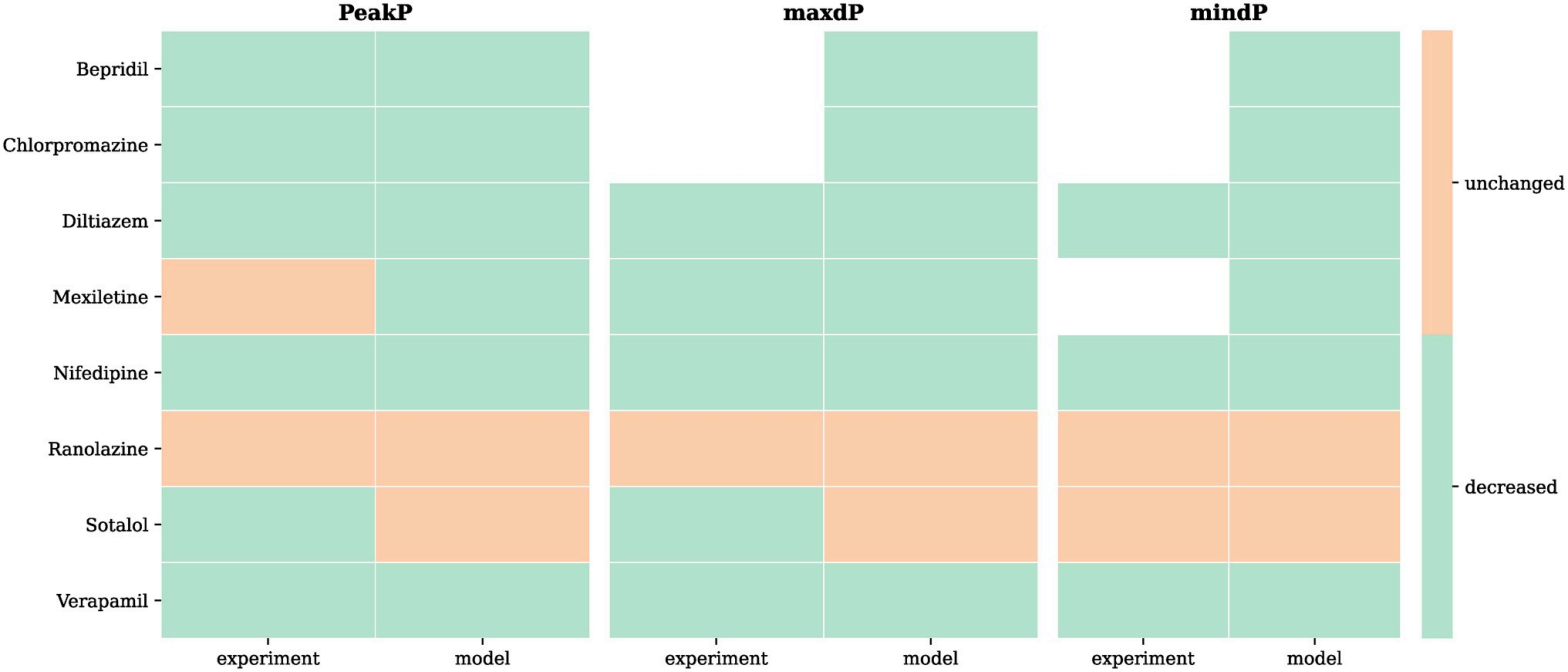
Model validation against known CiPA compounds effects on whole-organ function. Eight CiPA compounds effects (“experiment” columns) on PeakP, maxdP and mindP are compared with model same compounds’ predicted effects on the same features (“model” columns). The effects of compounds are colour-coded as orange (feature unchanged) and green (feature decreased). None of the compounds caused an increase in the considered LV features. White/empty space means that the specific effect could not be retrieved from the examined literature studies. Model predicted effects are in agreement with the experimentally observed effects for 6 out of 8 compounds.

A comparison of qualitative model predictions against experimental observations is shown in Fig 4. The model correctly predicted 16 out of 19 (84%) experimental observations, with 5 observations missing data, matching 6 out of 8 compounds. Model predictions were never opposite to observations, with either the model or observations reporting no-change when the model failed. A quantitative comparison was also performed when data was available. This is provided in S4 Text.

### 3.3 Building a model of the obese ZSF1 rat

**Fig 5:**
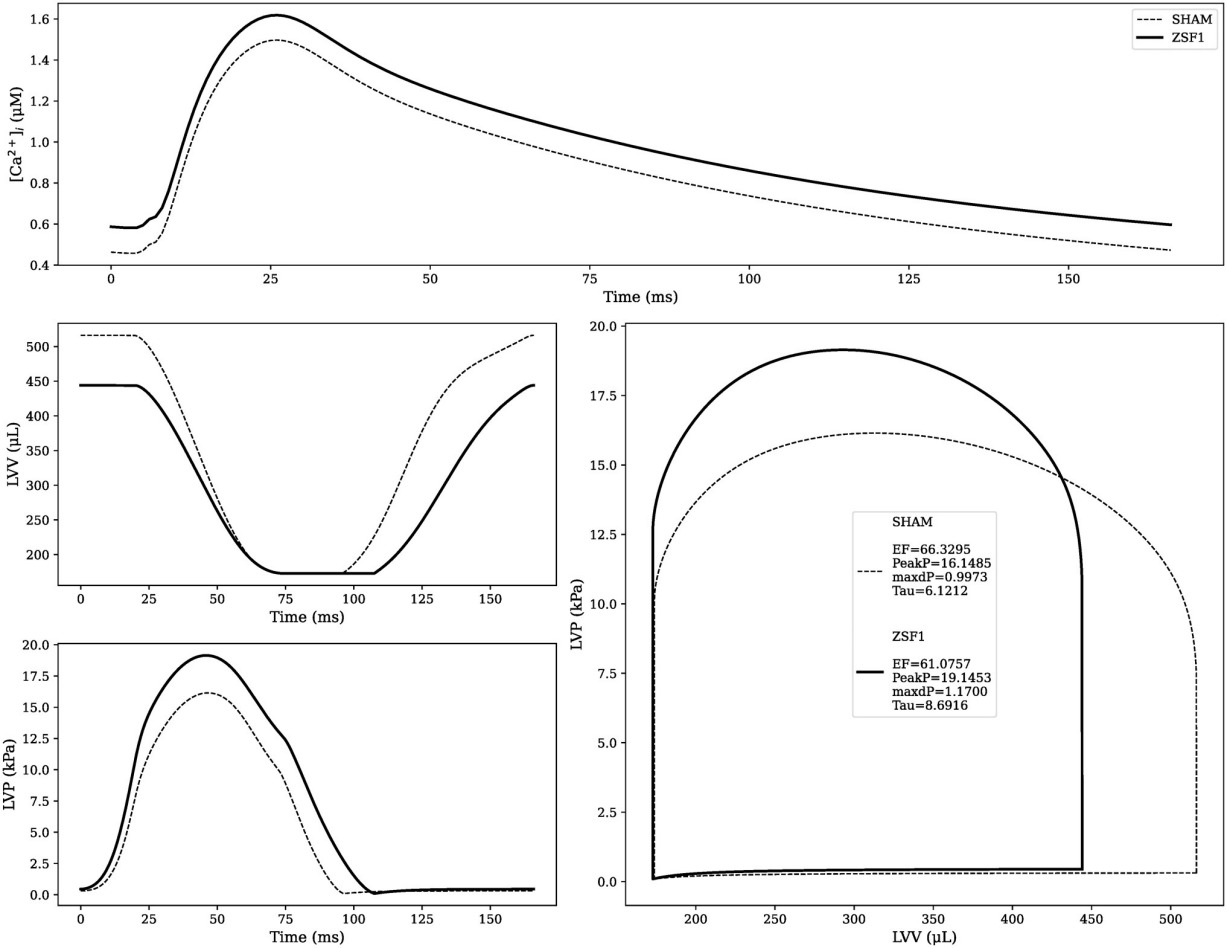
Representative SHAM and ZSF1 rat models calcium transients and pressure-volume loops. The ZSF1 rat model is created by perturbing the SHAM, healthy state. LV features which significantly changed from control to diseased animal are highlighted at the center of the PV loops sub-plot. EF showed no significant change.

In order to create a mathematical model of the obese ZSF1 rat, we performed a literature search to characterise the experimentally observed variability on LV systolic and diastolic function this rat shows with respect to its control animal. We than applied this variability to our SHAM control rat model, and we re-fitted model parameters using the HM technique (Section 2.3), trying to match the calculated shift in the LV function in the ZSF1 rats. The obtained representative ZSF1 rat model calcium transient and PV loop are depicted in Fig 5, and are compared with the reference SHAM rat model. Details of each step in the ZSF1 rat model creation process, including the complete sets of re-fitted and fixed model parameters and corresponding LV features are reported in S5 Text.

### 3.4 *In silico* recovering HFpEF condition towards healthy condition

We aim to identify cellular properties that can be manipulated to recover the ZSF1 rat towards the SHAM rat within a purely virtual experiment. By “recover” we mean to bring the altered LV features’ values we observed for the ZSF1 rat model back to the values of the SHAM rat model. We will do so by specifically targeting different subsets of model parameters to represent *in silico* different possible pharmacological mechanisms of action.

To recover the ZSF1 rat we re-fitted different subsets of model parameters in the ZSF1 model to recover the SHAM model output features, within the certainty that these features are predicted by the model (S6 Text). The LV features we aimed to recover were EDV, ESV, PeakP, maxdP, Tau. The groups of parameters selected for optimisation were: calcium transient (Ca), thin filament (TNF), thick filament (TKF) (Table 3), and the three groups combined (CaMYO). For each group of *D* parameters **p** = (*p*_1_, …, *p*_*D*_) we first trained one univariate GPE for each of the target LV features to substitute the following deterministic map

$$\begin{array}{c} f:R^{D}\to R \end{array}$$ (12)

$$\begin{array}{c} p\mapsto f_{simul}\left(p,\;\left(p_{D+1}^{\text{ZSF}1},\ldots ,\;p_{16}^{\text{ZSF}1}\right)\right)=y \end{array}$$ (13)

with a probabilistic surrogate to predict the LV feature value y∈R for a given parameter set $p\in R^{D}$. This was done by sampling points (512 for Ca, TNF, TKF groups and 2048 for CaMYO group) from a Latin hypercube design over the restricted, *D*-dimensional parameter space and by running the full simulator *f*_*simul*_ at these points to build new training datasets while keeping all the remaining parameters $\left(p_{D+1}^{\text{ZSF}1},\ldots ,\;p_{16}^{\text{ZSF}1}\right)$ fixed to the ZSF1 reference values. The new training datasets had dimensions of 104, 129, 114 and 326 points for the Ca, TNF, TKF and CaMYO groups, respectively. The GPEs’ accuracy are reported in S6 Text for each group.

**Fig 6:**
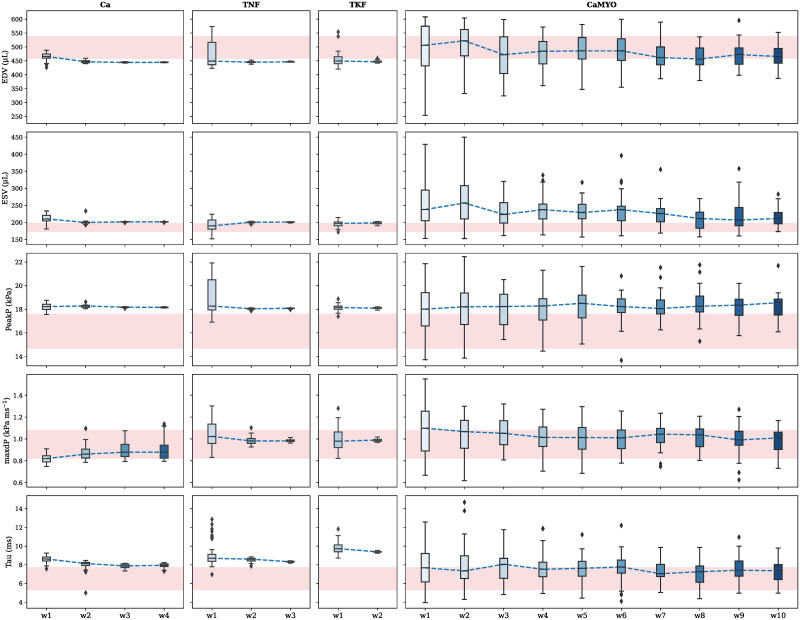
History matching waves progression. At each wave, 128 points are simulated from the current non-implausible parameter region and the features’ values from the converging simulations are plotted as box plots coloured in blue variants for different consecutive waves. The median trend of these distributions is represented by a dashed blue line. Mean ±3 standard deviations target intervals are represented in light red-coloured shaded areas for each LV feature.

For each parameter subgroup the adjustable parameters were re-fit using HM. Additional information about performed waves, used cutoff values and percentages of space reduction are reported in S6 Text. In Fig 6 the history matching waves progression is shown. For each LV feature we aimed to match, its values obtained when simulating parameter points from a specific wave’s non-implausible region are plotted as a distribution, possibly overlapping to the experimental variability (red band) observed for the same feature. This is done at every wave run, so that the HM is represented as a sequence of LV features values’ distributions over consecutive waves.

**Fig 7:**
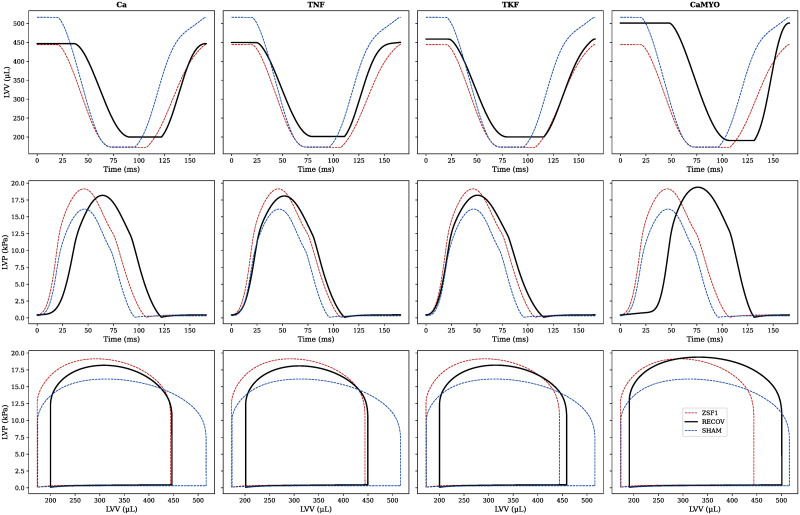
Best fit recovered rat heart models. For each parameter group, the last wave fitted models are compared against the target mean which the history matching aimed to match, and the best-fit model is selected according to the *L*_2_-norm. This best-fit rat model (RECOV, black thick line) is compared with the reference ZSF1 rat model (red dashed line) and with the reference SHAM rat model (blue dashed line).

For each parameter group, we can distinguish whether a specific LV feature has been recovered by looking at the last wave’ distribution. Specifically, a feature was determined to be recovered if this distribution median was within the uncertainty region for that feature. We can see that we were able to recover the maxdP feature in all 4 cases. EDV and Tau features were only recovered in the CaMYO group. The ESV feature was harder to recover, being very close to the uncertainty region in all the 4 cases although never meeting the median-based criterion. PeakP was never recovered, although it moved in the correct direction of recovery (decreasing) in all 4 cases. For each group, we selected (according to an *L*_2_-norm best-fit criterion) a reference recovered rat model which we labelled as “RECOV”, and we plotted the respective LV pressure and volume transients and PV loops (Fig 7), compared with the reference SHAM rat and ZSF1 rat models.

By relaxing the median-based recovery criterion, we also looked at whether the last wave’ distribution of a specific LV feature *i* had a median value $y_{i}^{\text{RECOV}}$ which was moving towards the healthy experimental mean value $y_{i}^{\text{SHAM}}$ starting from the reference, diseased state value $y_{i}^{\text{ZSF}1}$. For each parameter group, we computed the percentage of recovery *R*_perc_ for each LV feature as described by the ratio:

$$\begin{array}{c} R_{\text{perc}}=|\frac{y_{i}^{\text{RECOV}}-y_{i}^{\text{ZSF}1}}{y_{i}^{\text{SHAM}}-y_{i}^{\text{ZSF}1}}| \end{array}$$ (14)

A value of *R*_perc_ = 1 indicates that the feature has been recovered fully. When the median of a given LV feature’s distribution was not moving towards the correct direction of recovery, its corresponding *R*_perc_ value was set to 0. When the median was moving towards the correct direction of recovery but surpassed the healthy value, its corresponding *R*_perc_ value was set to 1 instead. *R*_perc_ values for each LV feature for each group are summarised in Table 4. We can see that the highest degree of recovery (39%) can be achieved when manipulating both the sarcomere kinetics and the calcium dynamics at the same time. It is worth noticing that the different degrees of recovery achieved by targeting the first three groups of parameters, namely 34%, 28%, 24% respectively for Ca, TNF, TKF groups, match the relative importance these groups have into explaining the total variance of the considered LV features (Table 3).

**Table 4:** LV features’ percentages of recovery. For each LV feature we aimed to recover, the distance between its median recovered value and the respective healthy value is divided by the distance between the initial, diseased value and the healthy value. This ratio describes the percentage of recovery for the examined feature.

LV feature	Parameter group			
	Ca	TNF	TKF	CaMYO
EDV	0.01	0.04	0.05	0.40
ESV	0.00	0.00	0.00	0.00
PeakP	0.33	0.36	0.35	0.20
maxdP	1.00	0.85	0.82	0.73
Tau	0.34	0.17	0.00	0.61
**Mean recovery**	0.34	0.28	0.24	0.39

**Fig 8:**
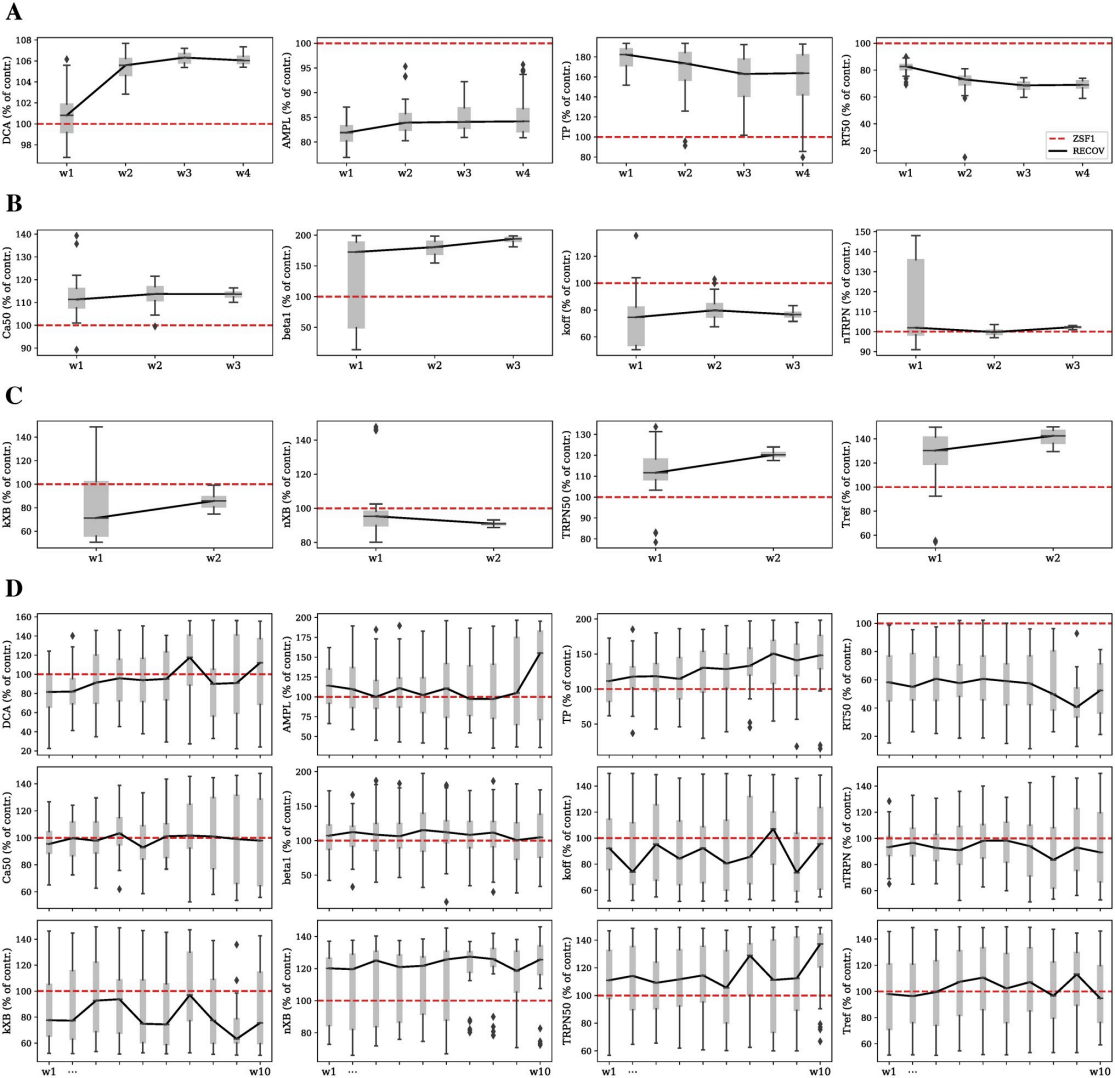
Parameter distributions across progressing waves for the four different parameter groups. Each parameter distribution is represented as a gray box plot at each wave. Its median trend during multiple waves is highlighted in a solid black line and is compared with the ZSF1 rat model reference value for the same parameter highlighted in a dashed red line. All the plotted RECOV parameter values are given as percentages of the respective baseline ZSF1 parameter values. (A) Ca group. (B) TNF group. (C) TKF group. (D) CaMYO group.

We further inspected the parameter space which the history matching converged to in the last wave for each parameter group, and we compared this with the ZSF1 rat model reference parameter set (see Fig 8). The distribution’s median trend over consecutive waves of each parameter within each group provides indication on which direction the parameter has undergone a perturbation from the reference ZSF1 rat same parameter value in order to recover the LV function. This is summarised in S6 Text for the last waves’ perturbations.

We can see that in order to recover the LV function by only perturbing the calcium transient (4 parameters), DCA and TP increased, while AMPL and RT50 decreased. This resulted in a calcium transient signal which was shifted upwards, flatter and delayed in time with fast recovery. By only perturbing the thin filament properties (4 parameters), the LV function could be recovered when Ca_50_ increased at a constant *n*_trpn_, with decreased *β*_1_ and *k*_off_. This resulted in TnC-Ca^2+^ bound complexes saturating at lower [Ca^2+^]_*i*_ and to a slower dissociation of the TnC-Ca^2+^ bound state which in turns made actin binding sites available for longer. By only perturbing the thick filament properties (4 parameters), the LV function could be recovered when *n*_xb_ and *k*_xb_ decreased with increased TRPN_50_ and *T*_ref_. This resulted in an overall slower force generation with increased maximal generated force. Lastly, when manipulating both the calcium transient and the whole sarcomere at the same time (12 parameters) to recover the LV function, AMPL and TP were increased at a constant DCA and decreased RT50; *k*_off_ and *n*_trpn_ were decreased at a constant Ca_50_ and *β*_1_; *n*_xb_ and TRPN_50_ were increased at a constant *T*_ref_ and decreased *k*_xb_.

**Fig 9:**
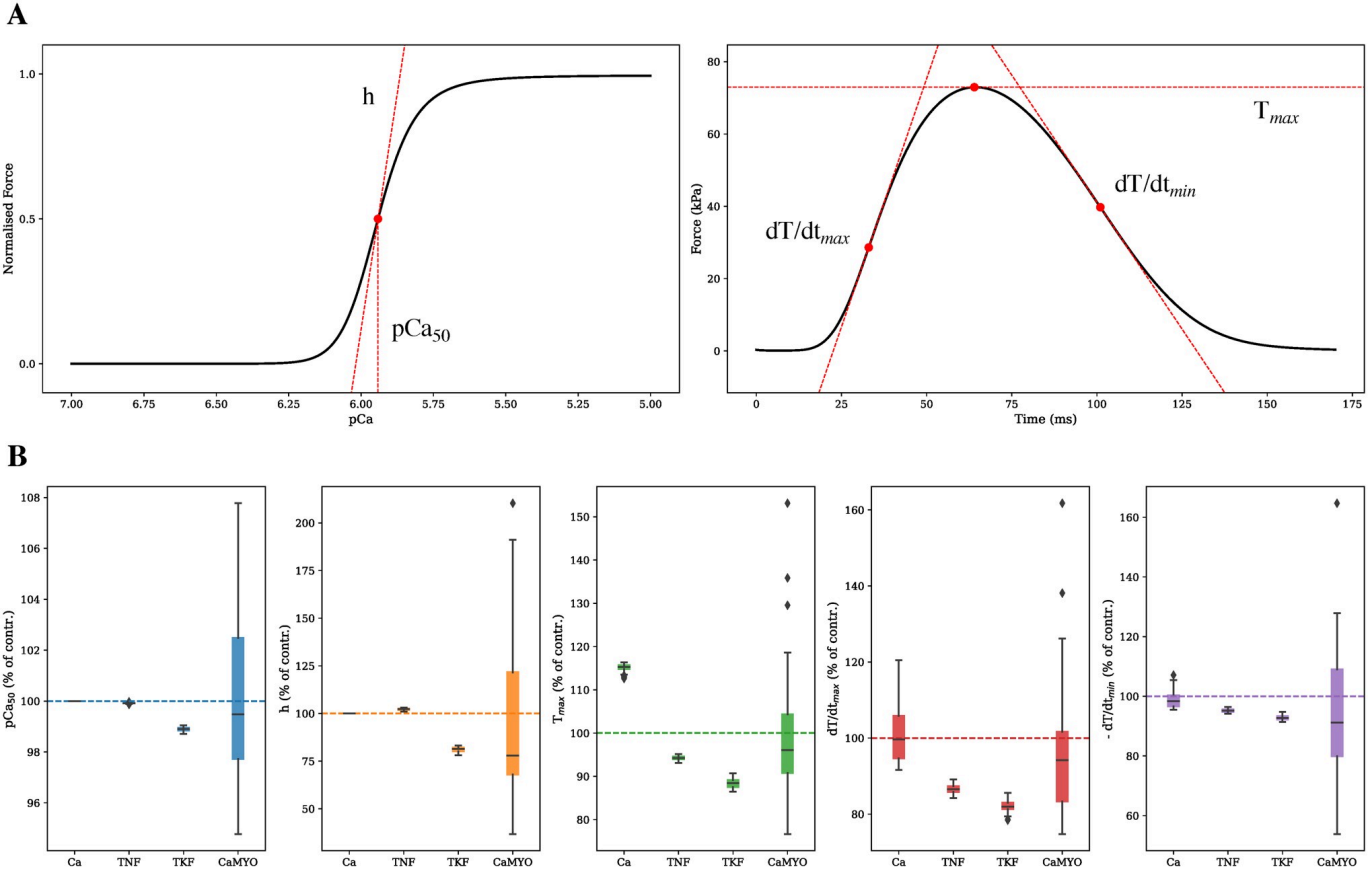
Isometric force-calcium relationship and generated active tension properties from the recovered rat model parameter space. (A) Calcium sensitivity (pCa_50_) and Hill coefficient (*h*) features are extracted from the force-calcium curve, while peak tension (T_*max*_) and maximum rates of tension development (dT/dt_*max*_) and decay (dT/dt_*min*_) are extracted from the twitch transient. (B) Distributions of extracted pCa_50_ (blue), *h* (orange), T_*max*_ (green), dT/dt_*max*_ (red), dT/dt_*min*_ (purple) values are compared with the respective ZSF1 rat model baseline values (dashed lines).

To interpret these changes in terms of intact muscle experimental measurements, we used the contraction model [6] to estimate the corresponding changes in steady state force-calcium relationship and field stimulated isometric tension transient predicted by the model to recover cardiac function in the ZSF1 model. This is illustrated in Fig 9. Common patterns can be observed in the way in which the four different simulated strategies of recovery act on the sarcomere. They all cause a none-to-rightwards shift of the force-calcium relationship, thereby desensitising the myofilament to intracellular calcium concentrations, and they all cause a no-change-to-decrease in the same curve’s Hill coefficient, resulting in an overall reduced affinity for calcium. Maximum generated active tension is always decreased apart from when the recovery is carried out via calcium transient modulation (Ca parameter group). Maximum rates of tension development and decay are always slowed down (less pronouncedly for the Ca strategy), which promoted the sarcomere to stay for longer in the force generating state.

## 4 Discussion

In this study, we proposed that calcium dynamics, thin and thick filament kinetics are all potential pharmacological targets for HFpEF, based on simulations in the ZSF1 rat model. The found recovered rat model parameter space, when interpreted in terms of muscle experimental measurements, also suggested that HFpEF-treating compounds should possibly act as direct sarcomere modulators by desensitising the myofilament and reducing the affinity to intracellular calcium, and decreasing the maximum generated active force while slowing down active force generation and relaxation in the intact muscle.

Previously, HFpEF was thought to result from solely diastolic dysfunction and LV hypertrophy [23]. However, therapies within this conceptual framework were not successful [24]. Recently, a combination of immune dysregulation and inflammation that leads to systemic microvascular endothelial dysfunction in various organ systems has been proposed as cause of HFpEF. There are now ∼20 pharmacotherapeutic clinical trials targeting signalling mechanisms along this cascade. Two of these respectively aim at blocking IL-1, a proinflammatory cytokine that inhibits the L-type calcium channels, and at inhibiting the late inward sodium current I_Na_. Both the therapies are expected to prevent cytosolic calcium overload, which may in turn improve LV relaxation (or *lusitropy*). This common end point is consistent with the targets identified in this study (Fig 8). Specifically, both the Ca and the CaMYO strategies of recovery proposed a decrease (of ∼30 − 50% from the diseased animal reference value) in the half-maximal calcium relaxation time, making less calcium available during the cardiac cycle. However, if this is accompanied by only a slight increase (∼5 − 10%) in the diastolic calcium concentration, a very prolonged (∼40 − 60%) time to peak calcium concentration is present as well, although this affects mostly the tension development and duration, rather then relaxation. We have seen that the Ca and CaMYO strategies have proposed a Ca^2+^ transient which is slower to rise and faster to decline, corresponding to a delayed and more symmetric Ca^2+^ transient. This causes a far greater delay between activation and contraction and a delayed systole, also visible in the left- and right-most panels in Fig 7. However, as more time is spent in isovolumetric contraction with unaltered ejection times, this results in negligible negative effects on the cardiac output. At the same time, this is accompanied by a shorter diastole, which in the case of HFpEF pathology constitutes an improvement for cardiac relaxation.

Targeting calcium handling is already subject of different clinical trials. On the other hand, the possibility to target the sarcomere to treat different cardiovascular pathologies including HFpEF by dynamically modulating its constituent proteins is an ongoing investigation [23]. Recent attempts of targeting the sarcomere have seen mavacamten and omecamtiv as protagonists. The first compound inhibits ATP hydrolysis thereby reducing myocardial contractility, while the second one activates cardiac myosin by stabilising it and favouring the power stroke, and it has been proposed as a treatment for HF with reduced EF. However, they are both indirect treatments for HFpEF, as in the first case only a chronic administration of mavacamten has been seen to reduce LV hypertrophy [25], while in the second case only in the presence of RV failure an HFpEF patient could benefit from increased RV contractility through omecamtiv action [26]. For this reason, the strategies of recovery proposed in this study by the TNF, TKF and CaMYO parameter groups cannot be directly compared to what is currently being tested experimentally (although we have already shown that single compounds’ effects can be quantitatively validated using mathematical models [27]), and therefore still miss thorough validation. As previous works (e.g. [28]) have demonstrated how models could be used for transferring findings between species, we don’t exclude the possibility for this framework to be scaled to human scale models, in order to potentially help the designing and developing of future diagnostic and therapeutic strategies.

### 4.1 Limitations

This work has a number of limitations. The model itself is a two-chamber simplification of a real heart, and spatial boundary conditions do not account for the pericardium which may have a role in constraining cardiac mechanics [29]. If the model is an approximation of the real system it represents, when we substitute it with an emulator we are adding an extra level of model discrepancy which will require further experts knowledge to be quantified. The performed GSA showed that altering preload and afterload has a secondary impact on the overall LV function. However, we modelled these two factors as fixed boundaries, and in more sophisticated closed loops heart systems the situation might change. The GSA also highlighted that parameters related to cross-bridge dynamics had a limited impact. This may be in part due to the length dependence of tension, where decreased *T*_ref_ or *k*_xb_ will lead to slower tension development, which will lead to the muscle remaining at higher sarcomere lengths for longer, and hence having higher Ca^2+^ sensitivity, which in turn will recover contraction. Additional *one-at-a-time* sensitivity analyses show (S7 Text) that, although *T*_ref_ has a non-negligible impact on e.g. ESV and SV over its full range of variability, this parameter has saturating effects at high levels on these features, whereas other parameters (e.g. calcium-related or thin filament-related) are operating at a maximum with reference parameter values and small changes will lead to a higher impact on the LV features. Similarly, the above discussed effects of changes in afterload and preload may be mitigated by the length dependence of tension. For model validation, most of the compounds we tested were calcium channel blockers, which are known to reduce the heart rate. Although this can be neutralised by secondary effects such as a reflex increase in beta adrenergic tone in response to systemic vascular dilation, so that because of the level of reflex beta adrenergic discharge the net effect on heart rate could be balanced out [30], our model doesn’t account for heart rate variations, so the validation was performed by evaluating the effects of compounds at a fixed, physiological rate. The negative chronotropic effect, coupled with the impact calcium channel blockers have on muscle sympathetic nerve activity [31], can also partially explain the mismatch observed (S4 Text) when quantitatively comparing compounds’ effects onto LV pressure features with the model simulations. Another source of mismatch can be linked to the fixed LV volume setup normally used in the considered experimental studies to evaluate the compounds’ effects, which differ from our rat heart model. The emulators were able to predict with high accuracy the same quantitative compounds’ effects on the examined LV features as the ones obtained using the simulator. However, the accuracy notably decreased and uncertainties increased when predicting high compound concentration values. We know that high concentrations of calcium channel blockers are associated with vanishing calcium transients. Since vanishing calcium transients also made the simulator fail due to small calcium signal amplitudes (S3 Text), the training dataset consequently did not contain parameter points encoding this kind of calcium transient shape. Therefore, predicting high compound concentration regions resulted in performing extrapolation outside the emulators’ training space boundaries, which can explain the observed reduction in prediction accuracy. Building a ZSF1 rat model by perturbing the SHAM model is a pragmatic choice. Ideally, one would want to start from MRI images of the obese ZSF1 rat and its related control (lean ZSF1 rat), create an *in silico* representation of both by fitting model parameters to hemodynamic measurements (possibly obtained from the same experimental rats cohorts) and then attempt to “virtually” recover the obese rat (diseased state) towards the lean rat (healthy state). The calculated percentages of recovery pointed out that all the parameter groups are able to recover cardiac function of similar degrees. Since every parameter group represents a strategy of recovery, this results in weighting all the types of recovery equally, and in real life situations each of them might have a different weight of clinical importance. However, this information can potentially be included in our analysis by weakening or strengthening the implausibility criterion for parameters that have to be more important than others.

## 5 Conclusion

We have used a validated biophysically detailed computational model of 3D biventricular rat heart mechanics and a Bayesian probabilistic framework to provide indication of potential cellular pharmacological targets to evaluate recovery of the LV function in an animal model of HFpEF. This combination of forward deterministic modelling with machine learning techniques proved to be crucial to carry out analysis which are normally too computational intensive to be performed within reasonable timescales. The developed framework can easily be adapted to solve many other different systems biology problems and could potentially aid the drug discovery and development process at preclinical stages.

## Supporting information

S1 Text Encoding calcium transient variations. (PDF)

S2 Text Model outputs and LV features extraction. (PDF)

S3 Text Gaussian process emulators. (PDF)

S4 Text Model validation. (PDF)

S5 Text Building a model of the obese ZSF1 rat. (PDF)

S6 Text *In silico* recovering HFpEF condition towards healthy condition. (PDF)

S7 Text Model response to input parameters’ variation. (PDF)
