# Supplementary material for: In silico identification of potential calcium dynamics and sarcomere targets for recovering left ventricular function in rat heart failure with preserved ejection fraction
Source: PLoS Comput Biol. 2021 Dec 6;17(12):e1009646. doi: 10.1371/journal.pcbi.1009646 (PMC8675924; doi:10.1371/journal.pcbi.1009646)
Supplement: S1 Text — (PDF) [file pcbi.1009646.s001.pdf]

S1 Encoding calcium transient variations

We used a 4-feature parametric representation of the calcium transient that maps to common experimentally measured features. The calcium transient was characterised using diastolic concentration (DCA), amplitude (AMPL), time to peak concentration (TP) and time to half-relaxation from peak concentration (RT50) features. We wish to sample random sets of these features such that the final samples will uniformly cover the feature space, in order to possibly cover both healthy and pathological calcium transient phenotypes. For this purpose, we used linear weighs to scale each of the four features from a representative calcium transient. This parametric encoding of calcium transient variation ensured that all transients maintained a characteristic calcium transient morphology.

The specific implementation of this scaling strategy is presented in Alg. 1. Note that `features` function returns the set of four features given an input calcium transient; `concatenate` function returns a 1D array obtained as the horizontal stack of the given input row 1D arrays; `linear_interpolator` function returns a function whose call method uses 1D linear interpolation to find the value of new points.

Algorithm 1 Scaling a representative calcium transient (y:=$[\text{Ca}^{2+}]_i(t)$) using linear interpolation.

Require: $t = (t_1, \ldots, t_N)$, $y = (y_1, \ldots, y_N)$, with $t_i, y_i \geq 0 \; \forall \; i = 1, \ldots, N$
$p = (p_1, \ldots, p_4)$, with $p_i \geq 0 \; \forall \; i = 1, \ldots, 4$
Ensure: $y_{new} \mid (\text{features}(t, y_{new}))_i = p_i \cdot (\text{features}(t, y))_i \quad \forall \; i = 1, \ldots, 4$
where features(t, y) = (DCA, AMPL, TP, RT50)

$\text{DCA} \leftarrow (\text{features}(t, y))_1$
$y_{new} \leftarrow p_1 \cdot \text{DCA} + p_2 \cdot (y - \text{DCA})$
$i_{max} \leftarrow \underset{i=1,\ldots,N}{\arg\max}\, y$
$T \leftarrow t_N$
if $p_3 \cdot (t_{i_{max}} - t_1) + p_4 \cdot (t_N - t_{i_{max}}) \leq T$ **then**
  $s \leftarrow (p_3 - p_4) \cdot t_{i_{max}}$
  $t_{tmp} \leftarrow \text{concatenate}(p_3 \cdot (t_1, \ldots, t_{i_{max}}),\ (p_4 \cdot t_{i_{max}+1} + s, \ldots, p_4 \cdot t_{N-1} + s),\ (t_N))$
  $f \leftarrow \text{linear\_interpolator}(t_{tmp}, y_{new})$
  $y_{new} \leftarrow f(t)$
else
  print "Not a valid scaling! Returning original calcium curve."
  $y_{new} \leftarrow y$
end if
return y_{new}

Fig S1.1 shows how the algorithm works and how new random calcium transients can be generated.

An important property of this calcium scaling strategy is that the features scale linearly with the scaling coefficient used, as shown in Fig S1.2. This allows us to randomly sample the scalar parameters that encode the calcium transient from a space filling design (e.g. a Latin hypercube) while generating samples that cover the full feature space. Having a training dataset with input parameters that uniformly cover the high-dimensional parameter space also helps Gaussian process emulators training while at the same time improving their prediction accuracy.

Fig S1.1. A 4-feature parametric representation of the calcium transient. (A.1) The calcium transient is described by four relevant quantities: diastolic concentration (DCA), amplitude (AMPL), time to peak concentration (TP) and time to half-relaxation from peak concentration (RT50). (B.1–4) Each of the 4 calcium features can be scaled independently to produce a new calcium transient. (A.2) All the features can be scaled at the same time to produce many different new calcium transients.

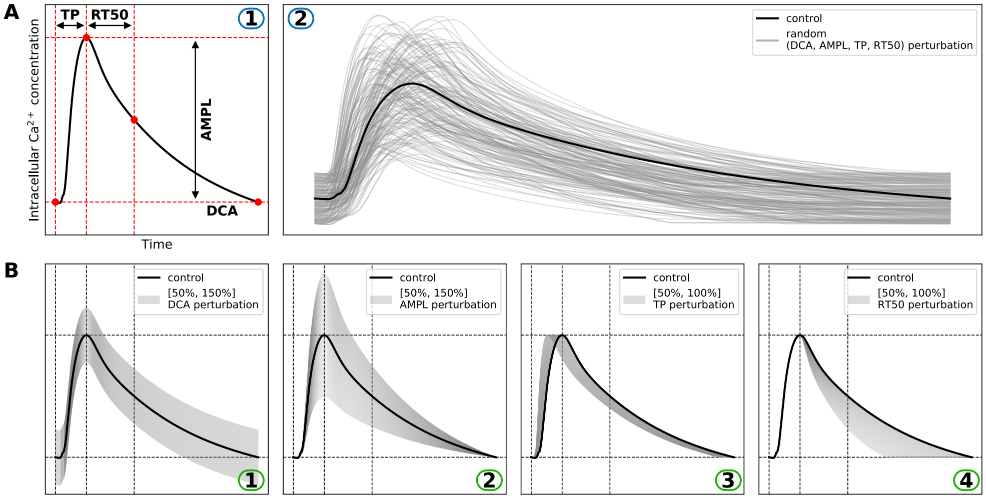

Fig S1.2. Calcium transient features linearly scale with their respective scaling coefficients. Example showing [50 %, 150 %] perturbation for all the calcium features but RT50, which undergoes a [50 %, 110 %] perturbation.

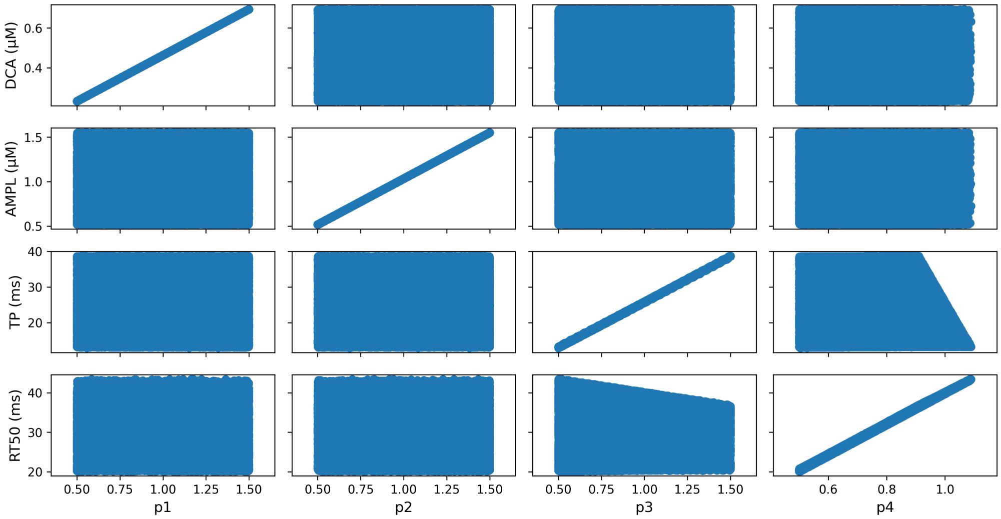

The employed ionic model generates a calcium transient for a fixed pacing rate (6 Hz), so that it occurs within a fixed time span. At physiological pacing rates, the rat calcium transient never reaches an equilibrium in diastole, so that during a calcium transient time is spent rising or relaxing. As the cell is paced at a fixed cycle length, the sum of time to peak and relaxation time is capped. This means that one cannot increase without the other decreasing, and independent perturbation of TP and RT50

can only decrease. If relaxation does interdependently slow, this will mean that the cell will not have enough time to fully relax, and this effect is captured by elevating DCA while decreasing AMPL. From an algorithmic view point, not all the randomly picked calcium features' scaling coefficient sets will therefore be viable. The "if" statement in Alg. 1 controls this behaviour by discarding all those curves that decay outside the fixed time interval without fully repolarising.

The existing coupling between TP and RT50 calcium features manifests as regions of the feature space that can not be captured by our calcium transient scaling strategy. These are indiecated by the blank triangels in the p_3-vs-RT50 and p_4-vs-TP subplots, visible in Fig S1.2.
