# Supplementary material for: In silico identification of potential calcium dynamics and sarcomere targets for recovering left ventricular function in rat heart failure with preserved ejection fraction
Source: PLoS Comput Biol. 2021 Dec 6;17(12):e1009646. doi: 10.1371/journal.pcbi.1009646 (PMC8675924; doi:10.1371/journal.pcbi.1009646)
Supplement: S2 Text — (PDF) [file pcbi.1009646.s002.pdf]

S2 Model outputs and LV features extraction

We characterised the LV function in both healthy and diseased biventricular rat heart models using 14 scalar quantities of clinical interest (Table 2 of the main manuscript). These quantities were extracted from the full multi-scale rat heart contraction model output, which is given as the LV volume and LV pressure transients during the last beat of a 4-beat long simulation.

Fig S2.1. LV output features extraction. The rat heart contraction model is run for a specific input parameters set for 4 heart beats. The 14 features of interest are then extracted from the fourth beat's LV volume and pressure curves.

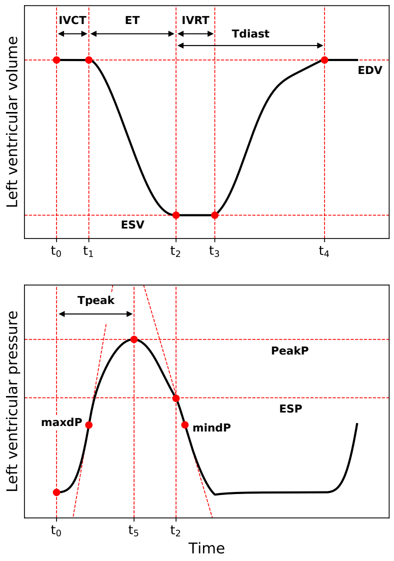

$$\text{EDV} = \max_{t>0} V(t) \tag{1}$$

$$\text{ESV} = \min_{t>0} V(t) \tag{2}$$

$$\text{SV} = \text{EDV} - \text{ESV} \tag{3}$$

$$\text{EF} = 100 \times \frac{\text{SV}}{\text{EDV}} \tag{4}$$

$$\text{IVCT} = t_1 - t_0 \tag{5}$$

$$\text{ET} = t_2 - t_1 \tag{6}$$

$$\text{IVRT} = t_3 - t_2 \tag{7}$$

$$\text{Tdiast} = t_4 - t_2 \tag{8}$$

$$\text{PeakP} = \max_{t>0} P(t) = P(t_5) \tag{9}$$

$$\text{Tpeak} = \arg\max_{t>0} P(t) = t_5 \tag{10}$$

$$\text{ESP} = P(t_2) \tag{11}$$

$$\text{maxdP} = \max_{t>0} \frac{dP(t)}{dt} \tag{12}$$

$$\text{mindP} = \min_{t>t_2} \frac{dP(t)}{dt} \tag{13}$$

$$\text{Tau} = -\frac{\min_{t>t_2} P(t) - P\left(\arg\min_{t>t_2} \frac{dP(t)}{dt}\right)}{2 \times \text{mindP}} \tag{14}$$
