# Supplementary material for: In silico identification of potential calcium dynamics and sarcomere targets for recovering left ventricular function in rat heart failure with preserved ejection fraction
Source: PLoS Comput Biol. 2021 Dec 6;17(12):e1009646. doi: 10.1371/journal.pcbi.1009646 (PMC8675924; doi:10.1371/journal.pcbi.1009646)
Supplement: S3 Text — (PDF) [file pcbi.1009646.s003.pdf]

S3 Gaussian process emulators

We employed Gaussian process emulators (GPEs) as probabilistic surrogates of the full multi-scale biventricular rat heart contraction model outputs (Section 2.1 of the main manuscript). This required first to define the high-dimensional input parameter space. Then it required to run simulations at parameter points sampled uniformly in this space such that the successfully completed simulation points could be used as GPEs' training dataset.

S3.1 Input parameter space

In Section 2.2 of the main manuscript, we have defined the high-dimensional input parameter space as the Cartesian product of individual, one-dimensional parameter ranges. Upper and lower bounds for these ranges were defined as percentages of the SHAM rat heart model reference parameters values. The adopted percentages of perturbation were chosen according to literature values from both modelling and experimental studies, and with the help of preliminary sensitivity analysis studies.

Specifically, for the calcium (Ca^{2+}) transient parameters/features (AMPL, DCA, TP and RT50) we performed a literature search to understand how these values could change when going from diseased to control animal in rat heart failure. We collected experimental observations from 30 experimental studies [1–30] on both HFrEF and HFpEF animal models, including AB (aortic-banded rat), TAC (transverse aortic constricted rat), CHF (rat with chronic heart failure), MI (rat with myocardial infarction), ZSF1 (Zucker diabetic fatty rat) DSS (Dhal salt-sensitive rat). To normalise observations across studies with different animal models and different pacing frequencies used for Ca^{2+} transient recording (0.5, 0.333, 1, 3, 4, 6, 7 Hz), we averaged mean percentage variations from control to diseased animals for each of the four Ca^{2+} transient parameters. Minimum and maximum percentage variations observed experimentally are reported as ranges in Table S3.1.

Table S3.1. Calcium transient parameters' experimental variability in heart failure rat models.

Parameter	Exp. variability (% of contr.)	Reference
DCA	[38.00 %, 200.0 %]	[1–30]
AMPL	[30.41 %, 203.23 %]	[1–30]
TP	[100.00 %, 151.26 %]	[1–30]
RT50	[59.56 %, 176.27 %]	[1–30]

The adopted range for the calcium parameters was [10 %, 200 %] of the respective control values, which achieved a full coverage of the experimentally observed variability (Table S3.1) while being nearly symmetric around the baseline value (100 %). The range for RT50 parameter was further adjusted to [10 %, 110 %] in order to limit the generation of implausible Ca^{2+} transients (where the sum of the time to peak and the relaxation time exceed the cycle length) when randomly scaling the reference Ca^{2+} transient using Algorithm 1 presented in S1 Text. Although the upper bound of this range seems not to cover the experimental observations fully, it is important to note that most heart failure studies are performed at non-physiological pacing rates (0.3-1 Hz), which allow much longer relaxation times that will not be seen at physiological (6 Hz) pacing rates. The relative distribution of experimental percentage variations values (blue box plot) is displayed overlapped to the chosen *in silico* range (blue shaded area) for each of the Ca^{2+} parameters in Fig S3.1.

Fig S3.1. Calcium transient parameters' experimental percentage variation distribution in heart failure rat models.

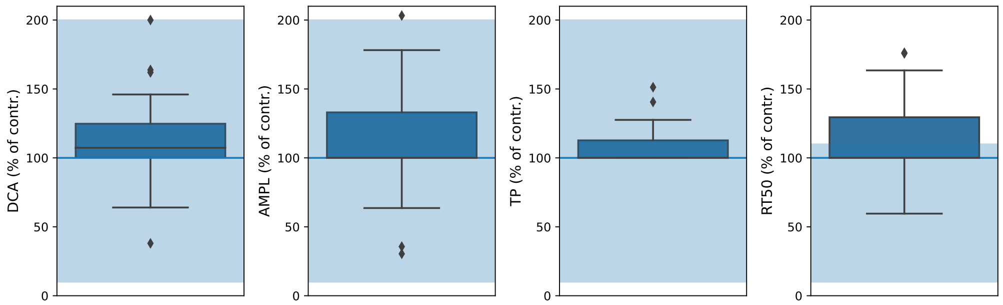

For the rest of the mechanics-regulating parameters (Ca_{50}, β_1, k_{off}, n_{trpn}, k_{xb}, n_{xb}, TRPN_{50}, T_{ref}, p, p_{ao}, Z, C_1) the adopted range was [50 %, 150 %] of the respective control values. This ensured that parameter values were consistent with the variability observed in both literature modelling and experimental studies on both healthy and diastolic heart failure rat models (Table S3.2), while being symmetric around the baseline value (100 %). Additional information from local sensitivity analysis studies was used to adjust the β_1 parameter range to be almost twice as big ([10 %, 200 %] of reference value), as LV features had a limited sensitivity to β_1 in the narrower range. The adopted ranges for the full set of simulator/emulator 16 input parameters are reported in Table S3.3.

Table S3.2. Mechanics-regulating parameters' values from experimental and modelling studies.

Parameter	Units	Value from experimental studies		Value from modelling studies	
		Range	Reference	Range	Reference
Ca_{50}	µM	–	–	[0.25, 3.25]	[31–37]
β_1	–	−1.5	[38,39]	[−4, −1.5]	[33,34,40]
k_{off}	ms^{-1}	[0.0013, 1.2]	[41–43]	[0.05, 0.2]	[31–34]
n_{trpn}	–	2	[44]	2	[34,40]
k_{xb}	ms^{-1}	0.1	[38,44]	[0.008, 0.2]	[32,33,33,40]
n_{xb}	–	5	[38,44]	[0.90, 7.05]	[40,45]
TRPN_{50}	–	0.35	[38]	[0.05, 0.50]	[40,45]
T_{ref}	kPa	–	–	[80, 202.89]	[31–34,44–51]
p	kPa	[0.2, 1.6]	[52–59]	[0.3, 1.4]	[32,33,40]
p_{ao}	kPa	[12, 26]	[52,56,57,59,59–63]	[6, 21]	[32,33,40]
Z	$\mathrm{mmHg\,s\,mL}^{-1}$	[1.5, 23]	[64–70]	[5.5, 20]	[31–33,71]
C_1	kPa	[0.1, 3.0]	[72]	[0.4, 1.6]	[31–33,46,73]

S3.2 Training dataset

In Section 2.2 of the main manuscript, we have seen that the training dataset in the 16-dimensional space was made of 1,299 points, which corresponded to only a small fraction (8.7 %) of the full, initial hypercube (14,848 simulated points, sampled uniformly using a Latin hypercube design). In Fig S3.2 we visually inspected the training dataset input parameter space to search for regions which could have been not well represented after training the emulators.

Fig S3.2 shows that there are regions of the input parameter space which are not

Table S3.3. Parameters' ranges used to construct the simulator/emulator 16-dimensional input parameter space.

Parameter	Units	Range
DCA	µM	[0.0463, 0.9264]
AMPL	µM	[0.1034, 2.0681]
TP	ms	[2.5947, 51.8947]
RT50	ms	[4.0081, 44.0888]
Ca_{50}	µM	[1.0861, 3.2584]
β_1	–	[−3.00, −0.15]
k_{off}	ms^{-1}	[0.0257, 0.0772]
n_{trpn}	–	[1.0, 3.0]
k_{xb}	ms^{-1}	[0.0086, 0.0258]
n_{xb}	–	[2.5, 7.5]
$TRPN_{50}$	–	[0.1750, 0.5250]
T_{ref}	kPa	[78.03, 234.10]
p	kPa	[0.1561, 0.4683]
p_{ao}	kPa	[3.5568, 10.6704]
Z	$\text{mmHg}\,\text{s}\,\text{mL}^{-1}$	[2.8117, 8.4351]
C_1	kPa	[0.4571, 1.3712]

Fig S3.2. Training dataset visual exploration. The GPEs' training dataset 16D input parameter space is plotted as a 2D projection for each pair of parameters (orange dots). The initial space (blue dots) simulated for building the training dataset is plotted in the same manner to highlight regions which are not covered by the training dataset.

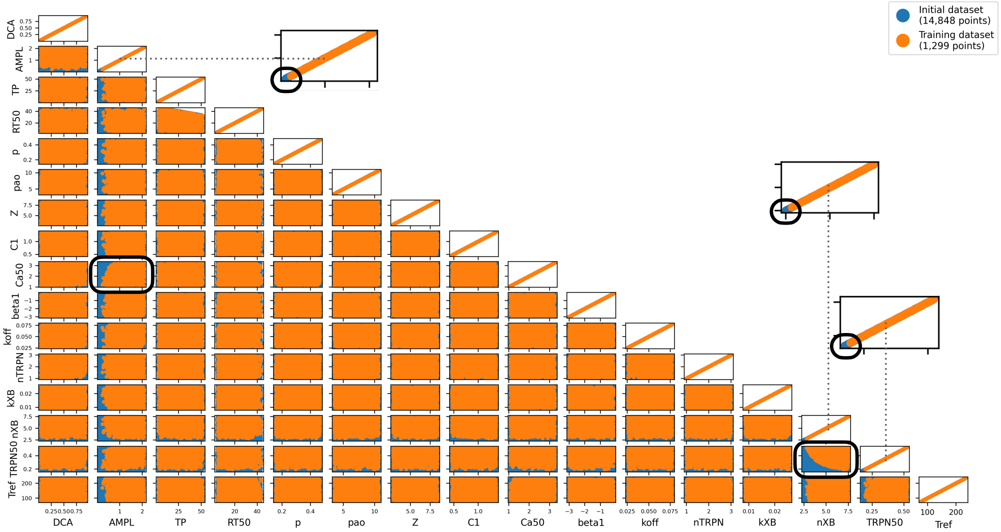

covered by the training dataset due to simulator failures at points from these regions. In particular, AMPL, n_{xb} and $TRPN_{50}$ parameters could not take values below 0.2, 2.8 and 0.195, respectively (simulated values were > 0.1, > 2.5 and > 0.175, respectively). This is consistently observed no matter the value of the other parameter points' components, concluding that there are 1-dimensional portions of the space which were not covered by the training dataset. 2-dimensional portions of the space which were not covered are also present. These involve AMPL-vs-Ca_{50} and n_{xb}-vs-$TRPN_{50}$ parameters' interactions. Specifically, low AMPL values could not co-exist with high Ca_{50} values, this is because high thin filament Ca^{2+} sensitivities rapidly activate the myofilament

but the small amount of available intracellular Ca^{2+} during systole is not enough to sustain contraction. Also, low n_{xb} values could not co-exist with low TRPN_{50} values, this is because they decrease the cross-bridges steady-state degree of cooperativity and sensitivity to bound Ca^{2+}-TnC complexes, making it hard for cross-bridges to form and to generate enough tension for the heart to contract.

In order to provide an estimate of the percentage area out of the total initial space area which was covered by the training dataset, we calculated for each parameter the percentage of final covered portion of its initial full 1D interval. We then multiplied all the percentage values obtained across the full set of input parameters, resulting in parameters covering $> 80\,\%$ of the full parameter space.

S3.3 Accuracy

A 5-fold cross-validation was used to quantify GPEs' accuracy by calculating the coefficient of determination (R^2) and the independent standard error (ISE_2), as described in Section 2.2 of the main manuscript.

Table S3.4. GPEs' accuracy. The GPEs' accuracy was evaluated using a 5-fold cross-validation. One fifth of the training dataset is held-out and one GPE is trained on the remaining points, for each LV feature. The left-out part is then used for testing the GPE accuracy, and an R^2 score and an ISE_2 are calculated. The process is repeated for each of the five subsets, randomly selected from the full training dataset. The final accuracy is determined by averaging the scores obtained in predicting the five different left-out parts.

LV feature	R^2	$\mathrm{ISE}_2(\%)$	LV feature	R^2	$\mathrm{ISE}_2(\%)$
EDV	0.94 ± 0.01	98.38 ± 0.61	Tdiast	0.50 ± 0.02	94.53 ± 1.07
ESV	0.83 ± 0.02	98.92 ± 0.75	PeakP	0.89 ± 0.01	98.61 ± 0.79
SV	0.79 ± 0.02	98.92 ± 0.66	Tpeak	0.49 ± 0.04	94.30 ± 1.15
EF	0.76 ± 0.01	98.77 ± 0.45	ESP	0.96 ± 0.01	98.07 ± 0.69
IVCT	0.52 ± 0.06	93.46 ± 1.19	maxdP	0.94 ± 0.01	98.46 ± 0.81
ET	0.79 ± 0.02	97.15 ± 1.49	mindP	0.90 ± 0.01	97.84 ± 1.10
IVRT	0.75 ± 0.05	97.54 ± 1.02	Tau	0.82 ± 0.02	96.69 ± 0.72

Fig S3.3. Inference using the best performing GPEs. For each LV feature, the GPE with the highest R^2 split test score is used to make predictions at the respective left-out subset of test points. Predictions are sorted in ascending order for the sake of a better visualisation and joined with a thick blue line, and the respective observations (empty dots) are sorted accordingly. 2 STD confidence intervals (shaded blue regions) are also plotted around predicted mean lines.

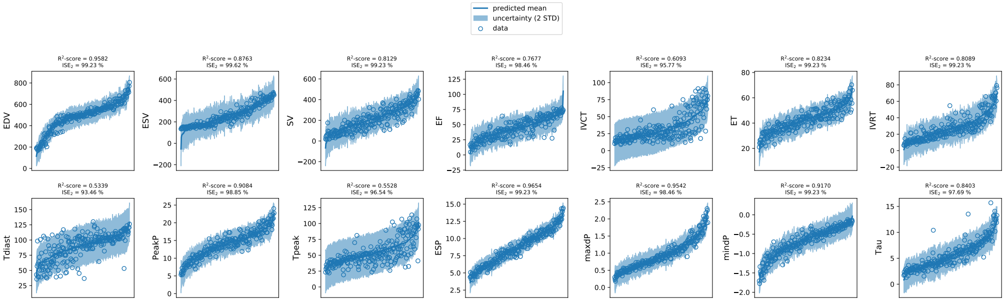

References

1. Abdellatif M, Trummer-Herbst V, Koser F, Durand S, Adão R, Vasques-Nóvoa F, et al. Nicotinamide for the treatment of heart failure with preserved ejection fraction. Sci Transl Med. 2021;13(580):eabd7064. doi:10.1126/scitranslmed.abd7064.

2. An S, Gilani N, Huang Y, Muncan A, Zhang Y, Tang YD, et al. Adverse transverse-tubule remodeling in a rat model of heart failure is attenuated with low-dose triiodothyronine treatment. Mol Med. 2019;25(1):1–14. doi:10.1186/s10020-019-0120-3.

3. Berni R, Savi M, Bocchi L, Delucchi F, Musso E, Chaponnier C, et al. Modulation of actin isoform expression before the transition from experimental compensated pressure-overload cardiac hypertrophy to decompensation. Am J Physiol - Hear Circ Physiol. 2009;296(5):1625–1632. doi:10.1152/ajpheart.01057.2008.

4. Bode D, Wen Y, Hegemann N, Primessnig U, Parwani A, Boldt LH, et al. Oxidative stress and inflammatory modulation of Ca2+ handling in metabolic HFPEF-related left atrial cardiomyopathy. Antioxidants. 2020;9(9):1–13. doi:10.3390/antiox9090860.

5. Bode D, Semmler L, Wakula P, Hegemann N, Primessnig U, Beindorff N, et al. Dual SGLT-1 and SGLT-2 inhibition improves left atrial dysfunction in HFpEF. Cardiovasc Diabetol. 2021;20(1):1–14. doi:10.1186/s12933-020-01208-z.

6. Call EMC, Ginsburg KS, Bassani Ra, Shannon TR, Qi M, Samarel AM, et al. Coupling in Hypertrophic Rat Ventricular Myocytes. Test. 1998;.

7. Chang KC, Figueredo VM, Schreur JHM, Kariya KI, Weiner MW, Simpson PC, et al. Thyroid hormone improves function and Ca2+ handling in pressure overload hypertrophy. Association with increased sarcoplasmic reticulum Ca2+-ATPase and α-myosin heavy chain in rat hearts. J Clin Invest. 1997;100(7):1742–1749. doi:10.1172/JCI119699.

8. Chen HH, Wang SN, Cao TT, Zheng JL, Tian J, Shan XL, et al. Stachydrine hydrochloride alleviates pressure overload-induced heart failure and calcium mishandling on mice. J Ethnopharmacol. 2020;248(October 2019):112306. doi:10.1016/j.jep.2019.112306.

9. Curl CL, Danes VR, Bell JR, Raaijmakers AJA, Ip WTK, Chandramouli C, et al. Cardiomyocyte functional etiology in heart failure with preserved ejection fraction is distinctive-a new preclinical model. J Am Heart Assoc. 2018;7(11). doi:10.1161/JAHA.117.007451.

10. van Deel ED, Najafi A, Fontoura D, Valent E, Goebel M, Kardux K, et al. In vitro model to study the effects of matrix stiffening on Ca2+ handling and myofilament function in isolated adult rat cardiomyocytes. J Physiol. 2017;595(14):4597–4610. doi:10.1113/JP274460.

11. Gattoni S, Røe ÅT, Aronsen JM, Sjaastad I, Louch WE, Smith NP, et al. Compensatory and decompensatory alterations in cardiomyocyte Ca 2+ dynamics in hearts with diastolic dysfunction following aortic banding. J Physiol. 2017;595(12):3867–3889. doi:10.1113/JP273879.

12. Hohendanner F, Bode D, Primessnig U, Guthof T, Doerr R, Jeuthe S, et al. Cellular mechanisms of metabolic syndrome-related atrial decompensation in a rat model of HFpEF. J Mol Cell Cardiol. 2018;115(November 2017):10–19. doi:10.1016/j.yjmcc.2017.12.012.

13. Hu ST, Tang Y, Shen YF, Ao HH, Bai J, Wang YL, et al. Protective effect of oxymatrine on chronic rat heart failure. J Physiol Sci. 2011;61(5):363–372. doi:10.1007/s12576-011-0154-y.

14. Ito N, Kagaya Y, Weinberg EO, Barry WH, Lorell BH. Endothelin and angiotensin II stimulation of Na+-H+ exchange is impaired in cardiac hypertrophy. J Clin Invest. 1997;99(1):125–135. doi:10.1172/JCI119123.

15. Kagaya Y, Weinberg EO, Ito N, Mochizuki T, Barry WH, Lorell BH. Glycolytic inhibition: Effects on diastolic relaxation and intracellular calcium handling in hypertrophied rat ventricular myocytes. J Clin Invest. 1995;95(6):2766–2776. doi:10.1172/JCI117980.

16. Kagaya Y, Hajjar RJ, Gwathmey JK, Barry WH, Lorell BH. Long-term angiotensin-converting enzyme inhibition with fosinopril improves depressed responsiveness to Ca2+ in myocytes from aortic-banded rats. Circulation. 1996;94(11):2915–2922. doi:10.1161/01.CIR.94.11.2915.

17. Kennedy D, Omran E, Periyasamy SM, Nadoor J, Priyadarshi A, Willey JC, et al. Effect of chronic renal failure on cardiac contractile function, calcium cycling, and gene expression of proteins important for calcium homeostasis in the rat. J Am Soc Nephrol. 2003;14(1):90–97. doi:10.1097/01.ASN.0000037403.95126.03.

18. Kilfoil PJ, Lotteau S, Zhang R, Yue X, Aynaszyan S, Solymani RE, et al. Distinct features of calcium handling and β-adrenergic sensitivity in heart failure with preserved versus reduced ejection fraction. J Physiol. 2020;598(22):5091–5108. doi:10.1113/JP280425.

19. Kim TY, Terentyeva R, Roder KHF, Li W, Liu M, Greener I, et al. SK channel enhancers attenuate Ca2+-dependent arrhythmia in hypertrophic hearts by regulating mito-ROS-dependent oxidation and activity of RyR. Cardiovasc Res. 2017;113(3):343–353. doi:10.1093/cvr/cvx005.

20. Loennechen JP, Wisløff U, Falck G, Ellingsen. Cardiomyocyte contractility and calcium handling partially recover after early deterioration during post-infarction failure in rat. Acta Physiol Scand. 2002;176(1):17–26. doi:10.1046/j.1365-201X.2002.01011.x.

21. Loennechen JP, Wisløff U, Falck G, Ellingsen Ø. Effects of cariporide and losartan on hypertrophy, calcium transients, contractility, and gene expression in congestive heart failure. Circulation. 2002;105(11):1380–1386. doi:10.1161/hc1102.105258.

22. Lyon AR, MacLeod KT, Zhang Y, Garcia E, Kanda GK, Lab MJ, et al. Loss of T-tubules and other changes to surface topography in ventricular myocytes from failing human and rat heart. Proc Natl Acad Sci U S A. 2009;106(16):6854–6859. doi:10.1073/pnas.0809777106.

23. Lyon AR, Bannister ML, Collins T, Pearce E, Sepehripour AH, Dubb SS, et al. SERCA2a gene transfer decreases sarcoplasmic reticulum calcium leak and reduces ventricular arrhythmias in a model of chronic heart failure. Circ Arrhythmia Electrophysiol. 2011;4(3):362–372. doi:10.1161/CIRCEP.110.961615.

24. Maçzewski M, Mackiewicz U. Effect of metoprolol and ivabradine on left ventricular remodelling and Ca2+ handling in the post-infarction rat heart. Cardiovasc Res. 2008;79(1):42–51. doi:10.1093/cvr/cvn057.

25. Maier LS, Brandes R, Pieske B, Bers DM. Effects of left ventricular hypertrophy on force and Ca2+ handling in isolated rat myocardium. Am J Physiol - Hear Circ Physiol. 1998;274(4 43-4). doi:10.1152/ajpheart.1998.274.4.h1361.

26. Meissner A, Min JY, Simon R. Effects of angiotensin II on inotropy and intracellular Ca2+ handling in normal and hypertrophied rat myocardium. J Mol Cell Cardiol. 1998;30(11):2507–2518. doi:10.1006/jmcc.1998.0813.

27. Min JY, Meissner A, Morgan JP. Mibefradil Improves β-drenergic Responsiveness and Intracellular Ca2+ Handling in Hypertrophied Rat Myocardium. Exp Biol Med. 2002;227(5):336–344. doi:10.1177/153537020222700506.

28. Miranda-Silva D, Wüst RCI, Conceição G, Gonçalves-Rodrigues P, Gonçalves N, Gonçalves A, et al. Disturbed cardiac mitochondrial and cytosolic calcium handling in a metabolic risk-related rat model of heart failure with preserved ejection fraction. Acta Physiol. 2020;228(3):1–17. doi:10.1111/apha.13378.

29. Rouhana S, Farah C, Roy J, Finan A, Rodrigues de Araujo G, Bideaux P, et al. Early calcium handling imbalance in pressure overload-induced heart failure with nearly normal left ventricular ejection fraction. Biochim Biophys Acta - Mol Basis Dis. 2019;1865(1):230–242. doi:10.1016/j.bbadis.2018.08.005.

30. Sadredini M, Danielsen TK, Aronsen JM, Manotheepan R, Hougen K, Sjaastad I, et al. Beta-adrenoceptor stimulation reveals Ca2+ waves and sarcoplasmic reticulum Ca2+ depletion in left ventricular cardiomyocytes from post-infarction rats with and without heart failure. PLoS One. 2016;11(4):1–18. doi:10.1371/journal.pone.0153887.

31. Land S, Niederer SA, Aronsen JM, Espe EKS, Zhang L, Louch WE, et al. An analysis of deformation-dependent electromechanical coupling in the mouse heart. J Physiol. 2012;590(18):4553–4569. doi:10.1113/jphysiol.2012.231928.

32. Lewalle A, Land S, Carruth E, Frank LR, Lamata P, Omens JH, et al. Decreasing compensatory ability of concentric ventricular hypertrophy in aortic-banded rat hearts. Front Physiol. 2018;9(FEB). doi:10.3389/fphys.2018.00037.

33. Longobardi S, Lewalle A, Coveney S, Sjaastad I, Espe EKS, Louch WE, et al. Predicting left ventricular contractile function via Gaussian process emulation in aortic-banded rats. Philos Trans A Math Phys Eng Sci. 2020;378(2173):20190334. doi:10.1098/rsta.2019.0334.

34. Niederer SA, Hunter PJ, Smith NP. A quantitative analysis of cardiac myocyte relaxation: A simulation study. Biophys J. 2006;90(5):1697–1722. doi:10.1529/biophysj.105.069534.

35. Wei Dong Gao, Backx PH, Azan-Backx M, Marban E. Myofilament Ca2+ sensitivity in intact versus skinned rat ventricular muscle. Circ Res. 1994;74(3):408–415. doi:10.1161/01.res.74.3.408.

36. Gao WD, Atar D, Backx PH, Marban E. Relationship Between Intracellular Calcium and Contractile Force in Stunned Myocardium. Circ Res. 1995;76(6):1036–1048. doi:10.1161/01.res.76.6.1036.

37. Backx PH, Gao WD, Azan-Backx MD, Marban E. The relationship between contractile force and intracellular [Ca2+] in intact rat cardiac trabeculae. J Gen Physiol. 1995;105(1):1–19. doi:10.1085/jgp.105.1.1.

38. Stull LB, Leppo MK, Marbán E, Janssen PML. Physiological Determinants of Contractile Force Generation and Calcium Handling in Mouse Myocardium. J Mol Cell Cardiol. 2002;34(10):1367–1376. doi:10.1006/jmcc.2002.2065.

39. Stull LB, Hiranandani N, Kelley MA, Leppo MK, Marbán E, Janssen PML. Murine strain differences in contractile function are temperature- and frequency-dependent. Pflugers Arch Eur J Physiol. 2006;452(2):140–145. doi:10.1007/s00424-005-0020-y.

40. Land S, Niederer SA, Smith NP. Efficient computational methods for strongly coupled cardiac electromechanics. IEEE Trans Biomed Eng. 2012;59(5):1219–1228. doi:10.1109/TBME.2011.2112359.

41. Rosenfeld SS, Taylor EW. Kinetic studies of calcium binding to regulatory complexes from skeletal muscle. J Biol Chem. 1985;260(1):252–61.

42. Tikunova SB, Davis JP. Designing calcium-sensitizing mutations in the regulatory domain of cardiac troponin C. J Biol Chem. 2004;279(34):35341–35352. doi:10.1074/jbc.M405413200.

43. Davis JP, Norman C, Kobayashi T, Solaro RJ, Swartz DR, Tikunova SB. Effects of thin and thick filament proteins on calcium binding and exchange with cardiac troponin C. Biophys J. 2007;92(9):3195–3206. doi:10.1529/biophysj.106.095406.

44. Blanchard E, Seidman C, Seidman JG, LeWinter M, Maughan D. Altered crossbridge kinetics in the alphaMHC403/+ mouse model of familial hypertrophic cardiomyopathy. Circ Res. 1999;84(4):475–83. doi:10.1161/01.res.84.4.475.

45. Longobardi S, Sher A, Niederer SA. In Silico Mapping of the Omecamtiv Mecarbil Effects from the Sarcomere to the Whole-Heart and Back Again. In: Ennis DB, Perotti LE, Wang VY, editors. Functional Imaging and Modeling of the Heart. Cham: Springer International Publishing; 2021. p. 406–415.

46. Niederer SA, Smith NP. The Role of the Frank–Starling Law in the Transduction of Cellular Work to Whole Organ Pump Function: A Computational Modeling Analysis. PLoS Comput Biol. 2009;5(4):e1000371. doi:10.1371/journal.pcbi.1000371.

47. Stuyvers BD, McCulloch AD, Guo J, Duff HJ, Ter Keurs HEDJ. Effect of stimulation rate, sarcomere length and Ca2+ on force generation by mouse cardiac muscle. J Physiol. 2002;544(3):817–830. doi:10.1113/jphysiol.2002.024430.

48. Palmer BM, Fishbaugher DE, Schmitt JP, Wang Y, Alpert NR, Seidman CE, et al. Differential cross-bridge kinetics of FHC myosin mutations R403Q and R453C in heterozygous mouse myocardium. Am J Physiol Hear Circ Physiol. 2004;287:91–99. doi:10.1152/ajpheart.01015.2003.-The.

49. Kreutziger KL, Piroddi N, McMichael JT, Tesi C, Poggesi C, Regnier M. Calcium binding kinetics of troponin C strongly modulate cooperative activation and tension kinetics in cardiac muscle. J Mol Cell Cardiol. 2011;50(1):165–174. doi:10.1016/j.yjmcc.2010.10.025.

50. Rice JJ, Wang F, Bers DM, De Tombe PP. Approximate model of cooperative activation and crossbridge cycling in cardiac muscle using ordinary differential equations. Biophys J. 2008;95(5):2368–2390. doi:10.1529/biophysj.107.119487.

51. Bovendeerd PHM, Kroon W, Delhaas T. Determinants of left ventricular shear strain. Am J Physiol - Hear Circ Physiol. 2009;297(3):1058–1068. doi:10.1152/ajpheart.01334.2008.

52. Németh BT, Mátyás C, Oláh A, Lux Á, Hidi L, Ruppert M, et al. Cinaciguat prevents the development of pathologic hypertrophy in a rat model of left ventricular pressure overload. Sci Rep. 2016;6. doi:10.1038/srep37166.

53. Sato F, Isoyama S, Takishima T. Normalization of impaired coronary circulation in hypertrophied rat hearts. Hypertension. 1990;16(1):26–34. doi:10.1161/01.hyp.16.1.26.

54. Schunkert H, Weinberg EO, Bruckschlegel G, Riegger AJ, Lorell BH. Alteration of growth responses in established cardiac pressure overload hypertrophy in rats with aortic banding. J Clin Invest. 1995;96(6):2768–2774. doi:10.1172/JCI118346.

55. Liu J, Han P, Xiao Y, Liu J, James Kang Y, Jm L, et al. A novel knot method for individually measurable aortic constriction in rats. Am J Physiol Hear Circ Physiol. 2014;307:987–995. doi:10.1152/ajpheart.00990.2013.-A.

56. Ku HC, Su MJ. DPP4 deficiency preserved cardiac function in abdominal aortic banding rats. PLoS One. 2014;9(1). doi:10.1371/journal.pone.0085634.

57. Ruppert M, Korkmaz-Icöz S, Loganathan S, Jiang W, Lehmann L, Oláh A, et al. Pressure-volume analysis reveals characteristic sex-related differences in cardiac function in a rat model of aortic banding-induced myocardial hypertrophy. Am J Physiol Hear Circ Physiol. 2018;315:502–511. doi:10.1152/ajpheart.00202.2018.-Sex.

58. Schunkert H, Dzau VJ, Tang SS, Hirsch AT, Apstein CS, Lorell BH. Increased rat cardiac angiotensin converting enzyme activity and mRNA expression in pressure overload left ventricular hypertrophy. Effects on coronary resistance, contractility, and relaxation. J Clin Invest. 1990;86(6):1913–1920. doi:10.1172/JCI114924.

59. Ruppert M, Korkmaz-Icöz S, Li S, Németh BT, Hegedu ˝s P, Brlecic P, et al. Myocardial reverse remodeling after pressure unloading is associated with maintained cardiac mechanoenergetics in a rat model of left ventricular hypertrophy. Am J Physiol Hear Circ Physiol. 2016;311:592–603. doi:10.1152/ajpheart.00085.2016.-Pressure.

60. Kovács A, Oláh A, Lux Á, Mátyás C, Németh T, Kellermayer D, et al. Strain and strain rate by speckle-tracking echocardiography correlate with pressure-volume loop-derived contractility indices in a rat model of athlete's heart. J Physiol Hear Circ Physiol. 2015;308:743–748. doi:10.1152/ajpheart.00828.2014.-Contractile.

61. Lee K, Hwang HJ, Kim OS, Oh YJ. Assessment of dexmedetomidine effects on left ventricular function using pressure–volume loops in rats. J Anesth. 2017;31(1):18–24. doi:10.1007/s00540-016-2278-y.

62. Oláh A, Németh BT, Mátyás C, Horváth EM, Hidi L, Birtalan E, et al. Cardiac effects of acute exhaustive exercise in a rat model. Int J Cardiol. 2015;182(C):258–266. doi:10.1016/j.ijcard.2014.12.045.

63. Toledo C, Andrade DC, Lucero C, Arce-Alvarez A, Díaz HS, Aliaga V, et al. Cardiac diastolic and autonomic dysfunction are aggravated by central chemoreflex activation in heart failure with preserved ejection fraction rats. J Physiol. 2017;595(8):2479–2495. doi:10.1113/JP273558.

64. Kobayashi S, Yano M, Kohno M, Obayashi M, Hisamatsu Y, Ryoke T, et al. Influence of aortic impedance on the development of pressure-overload left ventricular hypertrophy in rats. Circulation. 1996;94(12):3362–8. doi:10.1161/01.cir.94.12.3362.

65. Levy BI, Michel JB, Salzmann JL, Azizi M, Poitevin P, Safar M, et al. Effects of chronic inhibition of converting enzyme on mechanical and structural properties of arteries in rat renovascular hypertension. Circ Res. 1988;63(1):227–239. doi:10.1161/01.RES.63.1.227.

66. Zuckerman BD, Yin FCP. Aortic impedance and compliance in hypertensive rats. Am J Physiol - Hear Circ Physiol. 1989;257(2). doi:10.1152/ajpheart.1989.257.2.h553.

67. Lin YT, Tseng YZ, Chang KC. Aminoguanidine Prevents Fructose-Induced Arterial Stiffening in Wistar Rats: Aortic Impedance Analysis. Exp Biol Med. 2004;229(10):1038–1045. doi:10.1177/153537020422901008.

68. Yin FCP, Spurgeon HA, Weisfeldt ML, Lakatta EG. Mechanical properties of myocardium from hypertrophied rat hearts. A comparison between hypertrophy induced by senescence and by aortic banding. Circ Res. 1980;46(2):292–300. doi:10.1161/01.RES.46.2.292.

69. Ioannou CV, Morel DR, Katsamouris AN, Katranitsa S, Startchik I, Kalangos A, et al. Left ventricular hypertrophy induced by reduced aortic compliance. J Vasc Res. 2009;46(5):417–425. doi:10.1159/000194272.

70. Chang RW, Chang CY, Wu MS, Yu HY, Luo JM, Chen YS, et al. Systolic aortic pressure-time area is a useful index describing arterial wave properties in rats with diabetes. Sci Rep. 2015;5(October):1–10. doi:10.1038/srep17293.

71. Westerhof N, Elzinga G. Normalized input impedance and arterial decay time over heart period are independent of animal size. Am J Physiol Integr Comp Physiol. 1991;261(1):R126–R133. doi:10.1152/ajpregu.1991.261.1.R126.

72. Nordbø Ø, Lamata P, Land S, Niederer S, Aronsen JM, Louch WE, et al. A computational pipeline for quantification of mouse myocardial stiffness parameters. Comput Biol Med. 2014;53:65–75. doi:10.1016/j.compbiomed.2014.07.013.

73. Omens JH, MacKenna DA, McCulloch AD. Measurement of strain and analysis of stress in resting rat left ventricular myocardium. J Biomech. 1993;26(6):665–676. doi:10.1016/0021-9290(93)90030-I.
