# Supplementary material for: In silico identification of potential calcium dynamics and sarcomere targets for recovering left ventricular function in rat heart failure with preserved ejection fraction
Source: PLoS Comput Biol. 2021 Dec 6;17(12):e1009646. doi: 10.1371/journal.pcbi.1009646 (PMC8675924; doi:10.1371/journal.pcbi.1009646)
Supplement: S4 Text — (PDF) [file pcbi.1009646.s004.pdf]

S4 Model validation

We validated the biventricular healthy rat heart contraction mechanics model by looking at the effects of 8 comprehensive in vitro proarrhythmia assay (CiPA) [1] compounds on calcium dynamics and by propagating these effects across scales through to LV altered pump function.

S4.1 CiPA compounds

Table S4.1. IC_{50} and Hill coefficient values describing the affinity of eight CiPA compounds with the I_{Na}, I_{to}, I_{K1} and I_{CaL} ion channels. The dash symbol indicates that the specific compound has no inhibitory effect on the respective ion channel. Values taken from [2, 3].

**Compound**	**Ion channel**			
	I_{Na}	I_{to}	I_{K1}	I_{CaL}
**bepridil**				
IC_{50} (nM)	$2.93 \cdot 10^3$	$8.59 \cdot 10^3$	-	$2.81 \cdot 10^3$
h	1.16	3.54	-	0.65
**chlorpromazine**				
IC_{50} (nM)	$4.54 \cdot 10^3$	$1.76 \cdot 10^7$	$9.27 \cdot 10^3$	$8.19 \cdot 10^3$
h	2.00	0.37	0.69	0.84
**diltiazem**				
IC_{50} (nM)	$1.11 \cdot 10^5$	$2.82 \cdot 10^9$	-	$1.12 \cdot 10^2$
h	0.70	0.17	-	0.71
**mexiletine**				
IC_{50} (nM)	-	-	-	$3.82 \cdot 10^4$
h	-	-	-	1.03
**nifedipine**				
IC_{50} (nM)	$2.84 \cdot 10^4$	-	-	$1.15 \cdot 10^1$
h	1.11	-	-	0.67
**ranolazine**				
IC_{50} (nM)	$6.88 \cdot 10^4$	-	-	-
h	1.42	-	-	-
**sotalol**				
IC_{50} (nM)	$1.14 \cdot 10^9$	$4.31 \cdot 10^7$	$3.05 \cdot 10^6$	$7.06 \cdot 10^6$
h	0.51	0.66	1.20	0.87
**verapamil**				
IC_{50} (nM)	-	$1.34 \cdot 10^4$	$3.49 \cdot 10^8$	$2.02 \cdot 10^2$
h	-	0.82	0.27	1.10

S4.2 Compounds effects on the Ca^{2+} transient

Fig S4.1. The effect of verapamil on intracellular calcium transient. Gattoni et al. [4] model is run using different ion channels conductances' scalers to simulate the effect of different concentrations (blue colour variants) of the example compound considered.

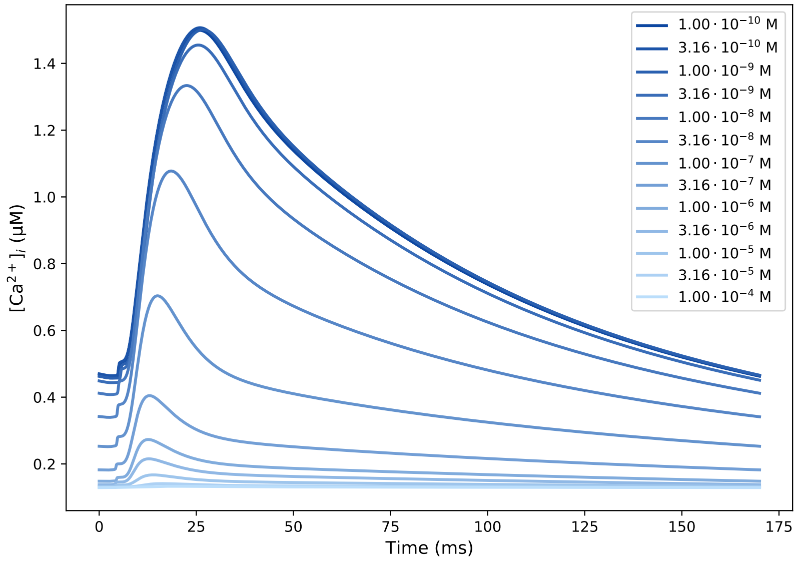

Fig S4.2. Calcium transient features dose-response curves. Calcium transient features are extracted from perturbed calcium transients and plotted against the respectively simulated compound concentrations.

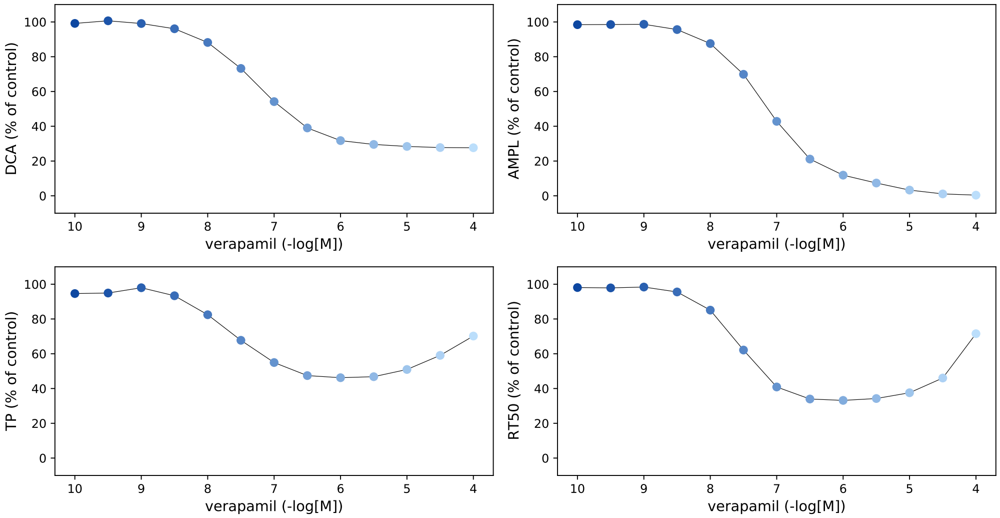

S4.3 Compounds effects on the LV function

Table S4.2. Qualitative change in three LV pressure features observed in literature rat experiments for eight different CiPA compounds. Down-facing arrow means that the specific LV feature decreases from its control value with the specific compound; left-right arrow means that the compound has no effect on that feature; dash symbol means that the specific information could not be retrieved from literature.

Compound	LV feature			References
	PeakP	maxdP	mindP	
bepridil	↓	–	–	[5–7]
chlorpromazine	↓	–	–	[8–10]
diltiazem	↓	↓	↓	[11–13]
mexiletine	↔	↓	–	[14–16]
nifedipine	↓	↓	↓	[13, 17, 18]
ranolazine	↔	↔	↔	[19–21]
sotalol	↓	↓	↔	[22–24]
verapamil	↓	↓	↓	[25–27]

Fig S4.3. LV pressure features' dose-response curves for the eight CiPA compounds. Simulated (dots in blue variants, colour-coded with the compound doses), emulated (full lines and shaded areas in blue) and experimentally observed (dots in red) PeakP, maxdP and mindP features' values are given as percentages of the respective control values. Experimental data taken from [6] (bepridil and verapamil – PeakP), [9] (chlorpromazine, PeakP), [12] (diltiazem – PeakP and maxdP), [17] (nifedipine – PeakP), [19] (ranolazine – PeakP, maxdP and mindP), [27] (verapamil – maxdP).

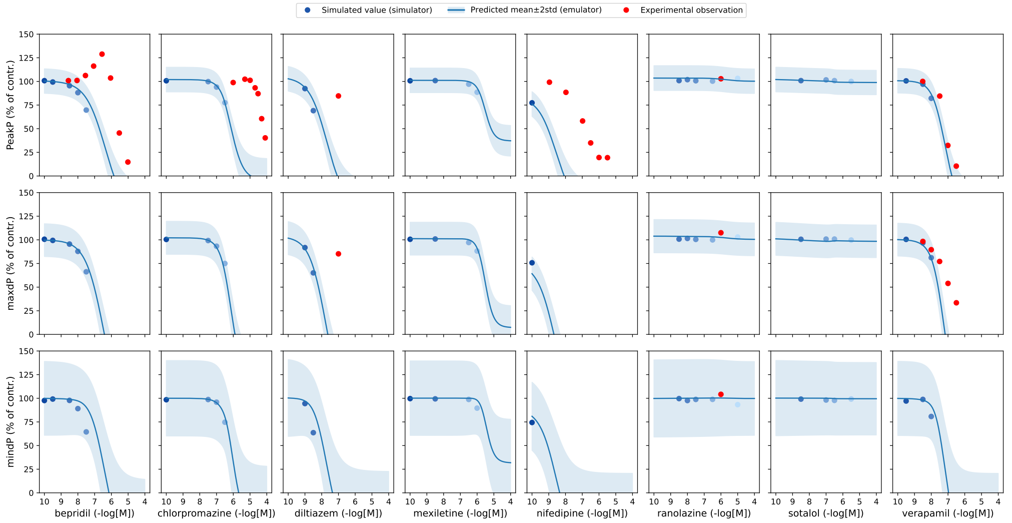

Fig S4.3 shows that both the model (simulator) and the surrogate model (emulator) predict the same LV features' trend of change from control in the presence of increasing compounds' concentrations. In the case of LV features' decreasing trends, the emulators' predictions were more accurate for lower compounds' concentrations than for higher compounds' concentrations, where we observed increased uncertainties and unphysiological (negative) predicted values (see Discussion of the main manuscript).

The simulated and predicted LV features' percentage changes were also compared with quantitative experimental measurements [6, 9, 12, 17, 19, 27] of compounds' effects when these were available (Table S4.2). Experimental data (highlighted in red in Fig S4.3) confirmed the trend (decreasing or unchanged) simulated/predicted by the simulator/emulator, although with a non-negligible mismatch in the absolute values (see Discussion of the main manuscript) for bepridil, chlorpromazine, diltiazem and nifedipine compounds (more pronounced) and ranolazine and verapamil compounds (mild).

The compounds' *in silico* effect on the pressure features of the right ventricle (RV) are reported as dose-response curves in Fig S4.4, and compared with the predicted response seen for the LV. We can see that the simulated change in function in the RV is consistent with the LV.

Fig S4.4. LV and RV pressure features' dose-response curves for the eight CiPA compounds. Simulated PeakP, maxdP and mindP features' values are given as percentages of the respective control values, and are represented as dots in blue/orange variants, colour-coded with the compound doses for respectively the left/right ventricles. Black dots indicate the control values when no compound is present.

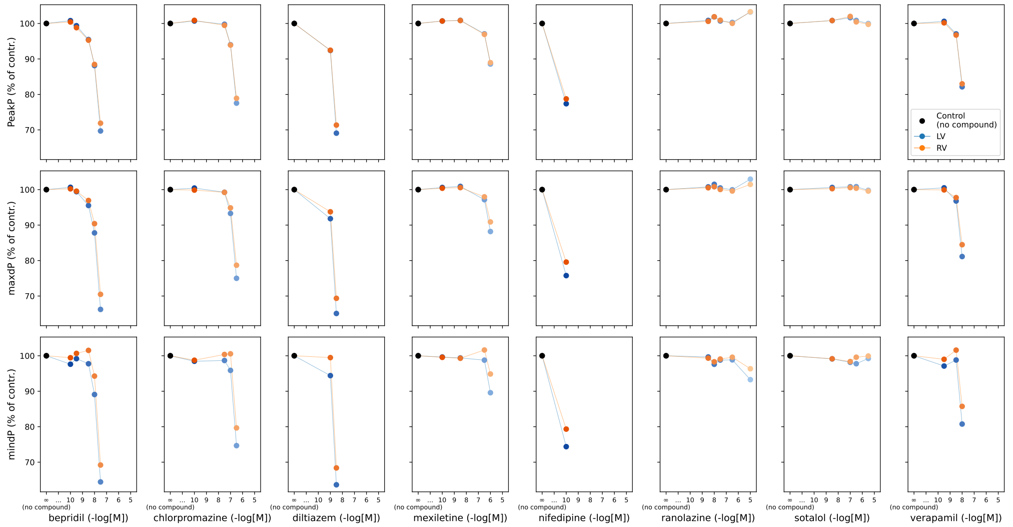

References

1. Park Js, Jeon Jy, Yang Jh, Kim Mg. Introduction to in silico model for proarrhythmic risk assessment under the CiPA initiative. Transl Clin Pharmacol. 2019;27(1):12–18.

2. Chang K, Li Z. FDA/CiPA; 2018. https://github.com/FDA/CiPA/blob/Model-Validation-2018/AP_simulation/data/newCiPA.csv.

3. Li Z, Ridder BJ, Han X, Wu WW, Sheng J, Tran PN, et al. Assessment of an In Silico Mechanistic Model for Proarrhythmia Risk Prediction Under the CiPA Initiative. Clin Pharmacol Ther. 2019;105(2):466–475. doi:10.1002/cpt.1184.

4. Gattoni S, Røe ÅT, Aronsen JM, Sjaastad I, Louch WE, Smith NP, et al. Compensatory and decompensatory alterations in cardiomyocyte Ca 2+ dynamics in hearts with diastolic dysfunction following aortic banding. J Physiol. 2017;595(12):3867–3889. doi:10.1113/JP273879.

5. de Leiris J, Harding DP, Pestre S. The isolated perfused rat heart: A model for studying myocardial hypoxia or ischaemia. Basic Res Cardiol. 1984;79(3):313–321. doi:10.1007/BF01908032.

6. van Amsterdam FTM, Zaagsma J. Stereoisomers of calcium antagonists discriminate between coronary vascular and myocardial sites. Naunyn Schmiedebergs Arch Pharmacol. 1988;337(2):213–219.

7. Huizer T, de Jong J, Achterberg P. Protection by bepridil against myocardial ATP-catabolism is probably due to negative inotropy. J Cardiovasc Pharmacol. 1987;10(1):55–61.

8. Katsuoka M, Ohnishi ST. Pharmacologic protection of perfused rat heart against global ischemia. Prostaglandins, Leukot Essent Fat Acids. 1989;38(3):151–156. doi:10.1016/0952-3278(89)90064-1.

9. Langslet A, Ryg M. Effects of Chlorpromazine and Propranolol on Left Ventricular Systolic Pressure, ECG, and K+ Efflux in the Isolated Perfused Rat Heart. Acta Pharmacol Toxicol (Copenh). 1971;29(5-6):533–541. doi:10.1111/j.1600-0773.1971.tb00627.x.

10. Sakai A, Sunada K. Effects of adrenaline on circulatory dynamics and cardiac function in rats administered chlorpromazine. Odontology. 2017;105(1):103–107. doi:10.1007/s10266-016-0241-x.

11. Flaim SF, Zelis R. Effects of diltiazem on total cardiac output distribution in conscious rats. J Pharmacol Exp Ther. 1982;222(2):359–366.

12. Koltai M, Tosaki A, Hosford D, Braquet P. Ginkgolide B protects isolated hearts against arrhythmias induced by ischemia but not reperfusion. Eur J Pharmacol. 1989;164(2):293–302. doi:10.1016/0014-2999(89)90470-6.

13. Dong H, Earle ML, Jiang Y, Loutzenhiser KA, Triggle CR. Cardiovascular effects of CPU-23, a novel L-type calcium channel blocker with a unique molecular structure. Br J Pharmacol. 1997;122(7):1271–1278. doi:10.1038/sj.bjp.0701508.

14. Kamiyama T, Tanonaka K, Harada H, Nakai K, Takeo S. Mexiletine and lidocaine reduce post-ischemic functional and biochemical dysfunction of perfused hearts. Eur J Pharmacol. 1995;272(2-3):151–158. doi:10.1016/0014-2999(94)00640-S.

15. Hesketh LM, Wilder CDE, Ranadive NN, Lytra G, Qazimi P, Munro JS, et al. Characterisation of mexiletine's translational therapeutic index for suppression of ischaemia-induced ventricular fibrillation in the rat isolated heart. Sci Rep. 2020;10(1):1–11. doi:10.1038/s41598-020-65190-y.

16. Marshall RJ, Muir AW, Winslow E. Mexiletine and Org 6001 in the Anaesthetized Rat. Br J Pharmacol. 1981;74:381–388.

17. Saponara S, Ferrara A, Gorelli B, Shah A, Kawase M, Motohashi N, et al. 3,5-Dibenzoyl-4-(3-phenoxyphenyl)-1,4-dihydro-2,6-dimethylpyridine (DP7): A new multidrug resistance inhibitor devoid of effects on Langendorff-perfused rat heart. Eur J Pharmacol. 2007;563(1-3):160–163. doi:10.1016/j.ejphar.2007.02.001.

18. Nishimura H, Kubota J, Okabe M, Ueyama M, Kawamura K. Nifedipine in divided doses does not reverse left ventricular hypertrophy in spontaneously hypertensive rats. Jpn Circ J. 1992;56(3):256–261.

19. Wang P, Fraser H, Lloyd SG, McVeigh JJ, Belardinelli L, Chatham JC. A comparison between ranolazine and CVT-4325, a novel inhibitor of fatty acid oxidation, on cardiac metabolism and left ventricular function in rat isolated perfused heart during ischemia and reperfusion. J Pharmacol Exp Ther. 2007;321(1):213–220. doi:10.1124/jpet.106.115519.

20. Hwang H, Arcidi JM, Hale SL, Simkhovich BZ, Belardinelli L, Dhalla AK, et al. Ranolazine as a cardioplegia additive improves recovery of diastolic function in isolated rat hearts. Circulation. 2009;120(SUPPL. 1):16–21. doi:10.1161/CIRCULATIONAHA.108.844167.

21. Wang GT, Li H, Yu ZQ, He XN. Effects of ranolazine on cardiac function in rats with heart failure. Eur Rev Med Pharmacol Sci. 2019;23(21):9625–9632. doi:10.26355/eurrev_201911_19456.

22. Mackin C, DeWitt ES, Black KJ, Tang X, Polizzotti BD, van den Bosch SJ, et al. Intravenous Amiodarone and Sotalol Impair Contractility and Cardiac Output, but Procainamide Does Not: A Langendorff Study. J Cardiovasc Pharmacol Ther. 2019;24(3):288–297. doi:10.1177/1074248418810811.

23. Hoffmeister HM, Seipel L. Comparison of the hemodynamics effects of D-sotalol and D,L-sotalol. Klin Wochenschr. 1988;66(10):451–454.

24. Lamontagne D, Rochette L, Vermeulen M, Yamaguchi N, Nadeau R, De Champlain J. Effect of sotalol against reperfusion-induced arrhythmias in Sprague-Dawley and Wistar rats. Fundamental & Clinical Pharmacology. 1989;3(6):671–685. doi:https://doi.org/10.1111/j.1472-8206.1989.tb00468.x.

25. Simonovic N, Jakovljevic V, Jeremic J, Finderle Z, Srejovic I, Nikolic Turnic T, et al. Comparative effects of calcium and potassium channel modulators on ischemia/reperfusion injury in the isolated rat heart. Mol Cell Biochem. 2019;450(1-2):175–185. doi:10.1007/s11010-018-3384-y.

26. Stojic I, Srejovic I, Zivkovic V, Jeremic N, Djuric M, Stevanovic A, et al. The effects of verapamil and its combinations with glutamate and glycine on cardiodynamics, coronary flow and oxidative stress in isolated rat heart. J Physiol Biochem. 2017;73(1):141–153. doi:10.1007/s13105-016-0534-0.

27. Kolář F, Ošt'ádal B, Papoušek F. Effect of verapamil on contractile function of the isolated perfused rat heart during postnatal ontogeny. Basic Res Cardiol. 1990;85(5):429–434. doi:10.1007/BF01931488.
