# Supplementary material for: In silico identification of potential calcium dynamics and sarcomere targets for recovering left ventricular function in rat heart failure with preserved ejection fraction
Source: PLoS Comput Biol. 2021 Dec 6;17(12):e1009646. doi: 10.1371/journal.pcbi.1009646 (PMC8675924; doi:10.1371/journal.pcbi.1009646)
Supplement: S5 Text — (PDF) [file pcbi.1009646.s005.pdf]

S5 Building a model of the obese ZSF1 rat

In order to create a mathematical model of the ZSF1 rat, we first needed to characterise its LV systolic and diastolic functions. For this purpose, we performed a literature search on PubMed (date 09/03/2021) with query: "(rat) AND (ZSF1) AND (hemodynamic)". The search gave 32 results and we analysed them all. Of these, 19 contained information about LV hemodynamic measurements. All the 19 studies [1–19] had been conducted on male rats at the age of 20 weeks, and diastolic dysfunction was confirmed by echocardiographic measurements (decreased E/A, increased E/E', increased LA area). We identified common phenotypes in these obese ZSF1 rats' hemodynamics compared to their respective control (lean ZSF1 rat) as described by 4 LV features, namely EF (no change), PeakP (increased), maxdP (increased), Tau (increased). To regularise observed changes in ZSF1 rats from multiple labs, we applied the observed average, percentage changes between ZSF1 rats and local control animals to our SHAM control rat model. The applied experimental variability is summarised in Table S5.1.

Table S5.1. Experimental ZSF1 obese rat hemodynamic data. For each LV feature of interest, mean and standard deviation values are given as percentages of the related control mean values.

LV feature	Exp. mean	Exp. std	Reference
EF	103.95 %	7.62 %	[2, 5, 7, 10–12, 15–17]
PeakP	119.26 %	6.07 %	[10, 12]
maxdP	125.46 %	5.26 %	[2, 7, 10, 11, 16]
Tau	141.97 %	13.67 %	[2, 5, 7, 10, 12, 15, 17]

To create a model of the ZSF1 rat, we re-fitted model parameters using the history matching (HM) technique (Section 2.3 of the main manuscript), trying to match the experimental variability shown for the above mentioned LV features. As EF did not change significantly in any experiment, we matched the EF feature value directly with no variability.

We considered the evidence for changing each of the 16 model parameters (Table 1 of the main manuscript). In ZSF1 rats, the intracellular calcium transient was shown to have increased diastolic concentrations and decreased/unaltered amplitudes at multiple frequencies (1-4 Hz-paced cells) [20, 21], so we fitted DCA and AMPL parameters. As the observed changes in time to peak calcium and time to calcium half-relaxation could not be extrapolated at physiological pacing rates, TP and RT50 parameters were kept fixed. Also, end-diastolic pressure [1, 2, 7–12, 15, 16] and cardiac tissue stiffness [1, 6, 7, 12, 13, 15, 16, 18] were consistently shown to be increased w.r.t control, so we fitted p and C_1 parameters. Furthermore, there was evidence for an increase in arterial systolic pressure [5] and aortic characteristic impedance [12], therefore parameters p_{ao}, Z were selected for optimisation as well. As there were no statistically significant changes in the reported values of myocardial active contraction in ZSF1 rats [22–24], we assumed that sarcomere properties remained unchanged (i.e. Ca_{50}, β_1, k_{off}, n_{trpn}, k_{xb}, n_{xb}, TRPN_{50}, T_{ref} parameters were not fitted). Although the ZSF1 rats all developed LV hypertrophy as shown by increased cardiac fibrosis, collagen type III fibers and cardiomyocyte size (increased LV mass and indexes of LV mass such as LV + IVS weights / tibial length), this was not always (e.g. [16, 18]) accompanied by an increase in LV wall thickness in male ZSF1 rats. Moreover, half of the ZSF1 rat studies [1, 7, 9–13, 18] showed no LV dilation as appraised by indexed end-diastolic volumes (preserved EDVi), measured by no change in the bi-ventricle size/shape. To summarise, 6 out of 16 SHAM rat model parameters were re-fitted to build the ZSF1 rat model while keeping the other parameters fixed to reference values.

The first wave of the HM procedure is displayed in Fig S5.1. We sampled 400,000 input parameter points from a Latin hypercube design over the parameter space *a priori* constrained by literature evidences (as explained above). These points were tested against an implausibility criterion with threshold set to 3.0 and 47,097 points (corresponding to 11.77 % of the full test set) were deemed non-implausible. The forward model was then run at a subset of 1024 non-implausible input parameter points to see if the respective simulation output was correctly matching the experimentally observed value for each LV feature under study. This is illustrated in Fig S5.2. Since all the simulated features' values already fell between 3 standard deviations from the respective experimental mean values, we concluded the HM.

Fig S5.1. First wave of history matching. The space represented by the parameters selected for optimisation is constrained according to an implausibility criterion which evaluates how plausible is a point to yield model predictions that are matching experimental observations.

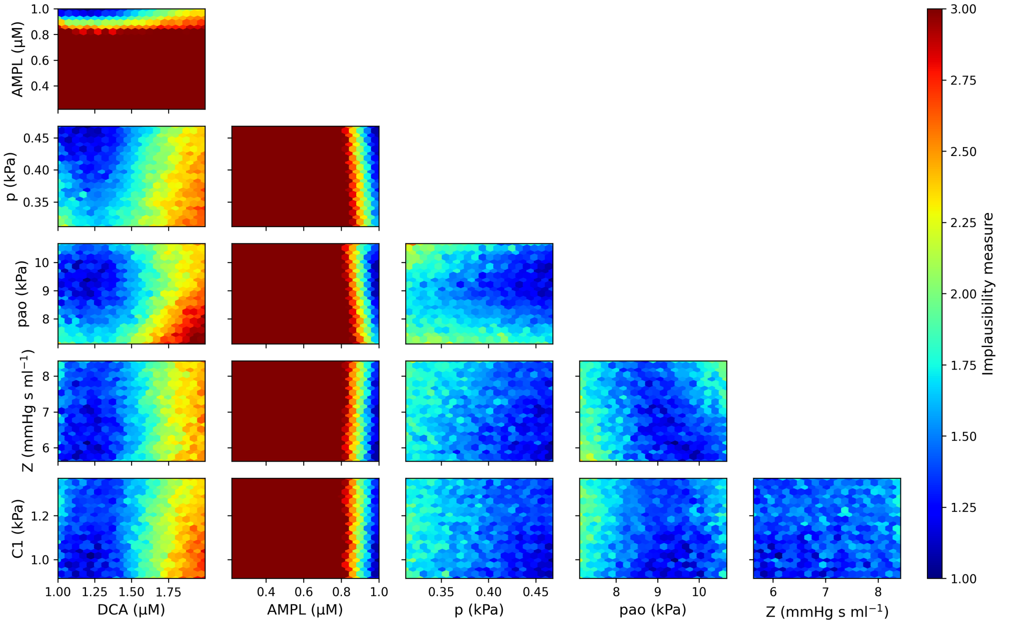

Unlike classical optimisation algorithms that converge to a single set of parameters possibly representing a global minimum of the loss function, the HM procedure proposes an entire cloud of points (belonging to the last wave's non-implausible, restricted parameter space) which are all possible candidates to yield a model features' match to experimental observation within experimental uncertainty. In order to have a representative ZSF1 rat model to be used in next investigations, we selected one candidate represented by the best-fit according to a weighted L_1 norm where the last three LV features (PeakP, maxdP and Tau) which were shown to significantly change in the ZSF1 rat had double the weight of the first feature (EF) which showed no significant change, and with all the weights summing up to 1. The best-fit ZSF1 rat model input calcium transient and PV loop are depicted in Fig 5 of the main manuscript, and are compared with the reference SHAM rat model. The complete sets of re-fitted and fixed model parameters and corresponding LV features for both the reference, control SHAM rat model and the newly obtained, representative ZSF1 rat model are reported in Table S5.2 and Table S5.3, respectively.

Fig S5.2. Matching experimental LV features' values. Simulator runs at input parameter points from the HM first (and also last) wave's non-implausible space. Obtained LV features' (empty, blue dots) distributions around experimental mean values (filled, black dots) are all within 3 STD confidence intervals (vertical straight lines centred in their respective mean value). The best fit in weighted L_1 norm is also displayed (black cross). All the values including confidence intervals have been normalised by the respective experimental mean values.

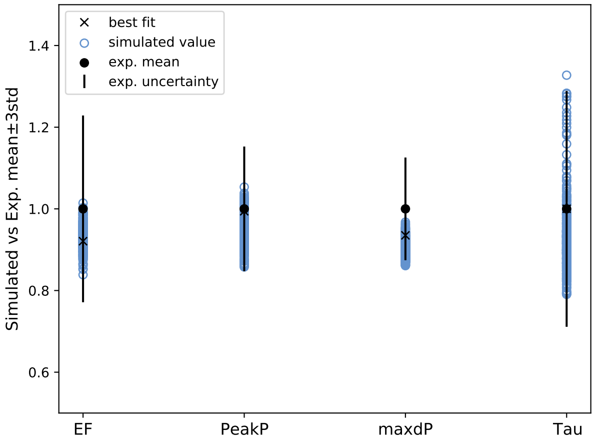

Table S5.2. Representative SHAM rat and ZSF1 rat models input parameters' values.

Parameter	Units	Value	
		**SHAM**	**ZSF1**
DCA	μM	0.4632	0.5870
AMPL	μM	1.0341	1.0317
TP	ms	25.9474	25.9474
RT50	ms	40.0807	40.0807
Ca_{50}	μM	2.1723	2.1723
β_1	–	−1.5	−1.5
k_{off}	ms^{-1}	0.0515	0.0515
n_{trpn}	–	2.0	2.0
k_{xb}	ms^{-1}	0.0172	0.0172
n_{xb}	–	5.0	5.0
$TRPN_{50}$	–	0.35	0.35
T_{ref}	kPa	156.067	156.067
p	kPa	0.3122	0.4481
p_{ao}	kPa	7.1136	10.3887
Z	mmHg s mL^{-1}	5.6234	7.4031
C_1	kPa	0.9141	1.0670

Table S5.3. Representative SHAM rat and ZSF1 rat models output LV features' values.

LV feature	Units	Value	
		**SHAM**	**ZSF1**
EDV	µL	516.23	444.02
ESV	µL	173.82	172.83
SV	µL	342.42	271.19
EF	%	66.33	61.08
IVCT	ms	19.3	19.7
ET	ms	52.9	54.6
IVRT	ms	23.8	33.0
Tdiast	ms	93.5	91.4
PeakP	kPa	16.15	19.15
Tpeak	ms	46.6	45.9
ESP	kPa	10.04	12.55
maxdP	$kPa\,ms^{-1}$	0.9973	1.1700
mindP	$kPa\,ms^{-1}$	-0.5712	-0.5276
Tau	ms	6.1212	8.6916

References

1. Abdellatif M, Leite S, Alaa M, Oliveira-Pinto J, Tavares-Silva M, Fontoura D, et al. Spectral transfer function analysis of respiratory hemodynamic fluctuations predicts end-diastolic stiffness in preserved ejection fraction heart failure. Am J Physiol - Hear Circ Physiol. 2016;310(1):H4–H13. doi:10.1152/ajpheart.00399.2015.

2. Bowen TS, Brauer D, Rolim NPL, Bækkerud FH, Kricke A, Ormbostad Berre AM, et al. Exercise training reveals inflexibility of the diaphragm in an animal model of patients with obesity-driven heart failure with a preserved ejection fraction. J Am Heart Assoc. 2017;6(10):1–16. doi:10.1161/JAHA.117.006416.

3. Bowen TS, Herz C, Rolim NPL, Berre AMO, Halle M, Kricke A, et al. Effects of Endurance Training on Detrimental Structural, Cellular, and Functional Alterations in Skeletal Muscles of Heart Failure With Preserved Ejection Fraction. J Card Fail. 2018;24(9):603–613. doi:10.1016/j.cardfail.2018.08.009.

4. Brandt MM, Nguyen ITN, Krebber MM, van de Wouw J, Mokry M, Cramer MJ, et al. Limited synergy of obesity and hypertension, prevalent risk factors in onset and progression of heart failure with preserved ejection fraction. J Cell Mol Med. 2019;23(10):6666–6678. doi:10.1111/jcmm.14542.

5. Cuijpers I, Carai P, Mendes-Ferreira P, Simmonds SJ, Mulder P, Miranda-Silva D, et al. The effect of different anaesthetics on echocardiographic evaluation of diastolic dysfunction in a heart failure with preserved ejection fraction model. Sci Rep. 2020;10(1):1–12. doi:10.1038/s41598-020-72924-5.

6. Davila A, Tian Y, Czikora I, Li J, Su H, Huo Y, et al. Adenosine kinase inhibition augments conducted vasodilation and prevents left ventricle diastolic dysfunction in heart failure with preserved ejection fraction. Circ Hear Fail. 2019;12(8):1–12. doi:10.1161/CIRCHEARTFAILURE.118.005762.

7. Hamdani N, Franssen C, Lourenço A, Falca o Pires I, Fontoura D, Leite S, et al. Myocardial titin hypophosphorylation importantly contributes to heart failure with preserved ejection fraction in a rat metabolic risk model. Circ Hear Fail. 2013;6(6):1239–1249. doi:10.1161/CIRCHEARTFAILURE.113.000539.

8. Hohendanner F, Bode D, Primessnig U, Guthof T, Doerr R, Jeuthe S, et al. Cellular mechanisms of metabolic syndrome-related atrial decompensation in a rat model of HFpEF. J Mol Cell Cardiol. 2018;115(November 2017):10–19. doi:10.1016/j.yjmcc.2017.12.012.

9. Lai YC, Tabima DM, Dube JJ, Hughan KS, Vanderpool RR, Goncharov DA, et al. SIRT3-AMP-Activated Protein Kinase Activation by Nitrite and Metformin Improves Hyperglycemia and Normalizes Pulmonary Hypertension Associated with Heart Failure with Preserved Ejection Fraction. Circulation. 2016;133(8):717–731. doi:10.1161/CIRCULATIONAHA.115.018935.

10. Leite S, Rodrigues S, Tavares-Silva M, Oliveira-Pinto J, Alaa M, Abdellatif M, et al. Afterload-induced diastolic dysfunction contributes to high filling pressures in experimental heart failure with preserved ejection fraction. Am J Physiol - Hear Circ Physiol. 2015;309(10):H1648–H1654. doi:10.1152/ajpheart.00397.2015.

11. Leite S, Oliveira-Pinto J, Tavares-Silva M, Abdellatif M, Fontoura D, Falcão-Pires I, et al. Echocardiography and invasive hemodynamics during stress

testing for diagnosis of heart failure with preserved ejection fraction: An experimental study. Am J Physiol - Hear Circ Physiol. 2015;308(12):H1556–H1563. doi:10.1152/ajpheart.00076.2015.

12. Leite S, Cerqueira RJ, Ibarrola J, Fontoura D, Fernández-Celis A, Zannad F, et al. Arterial Remodeling and Dysfunction in the ZSF1 Rat Model of Heart Failure With Preserved Ejection Fraction. Circ Hear Fail. 2019;12(7):1–12. doi:10.1161/CIRCHEARTFAILURE.118.005596.

13. Nguyen ITN, Brandt MM, van de Wouw J, van Drie RWA, Wesseling M, Cramer MJ, et al. Both male and female obese ZSF1 rats develop cardiac dysfunction in obesity-induced heart failure with preserved ejection fraction. PLoS One. 2020;15(5):1–16. doi:10.1371/journal.pone.0232399.

14. Park SH, Farooq MA, Gaertner S, Bruckert C, Qureshi AW, Lee HH, et al. Empagliflozin improved systolic blood pressure, endothelial dysfunction and heart remodeling in the metabolic syndrome ZSF1 rat. Cardiovasc Diabetol. 2020;19(1):1–14. doi:10.1186/s12933-020-00997-7.

15. Salah EM, Bastacky SI, Jackson EK, Tofovic SP. Captopril Attenuates Cardiovascular and Renal Disease in a Rat Model of Heart Failure with Preserved Ejection Fraction. J Cardiovasc Pharmacol. 2018;71(4):205–214. doi:10.1097/FJC.0000000000000561.

16. Schmederer Z, Rolim N, Bowen TS, Linke A, Wisloff U, Adams V. Endothelial function is disturbed in a hypertensive diabetic animal model of HFpEF: Moderate continuous vs. high intensity interval training. Int J Cardiol. 2018;273:147–154. doi:10.1016/j.ijcard.2018.08.087.

17. Stolina M, Luo X, Dwyer D, Han CY, Chen R, Zhang Y, et al. The evolving systemic biomarker milieu in obese ZSF1 rat model of human cardiometabolic syndrome: Characterization of the model and cardioprotective effect of GDF15. PLoS One. 2020;15(8 August):1–25. doi:10.1371/journal.pone.0231234.

18. Van Dijk CGM, Oosterhuis NR, Xu YJ, Brandt M, Paulus WJ, Van Heerebeek L, et al. Distinct endothelial cell responses in the heart and kidney microvasculature characterize the progression of heart failure with preserved ejection fraction in the obese ZSF1 rat with cardiorenal metabolic syndrome. Circ Hear Fail. 2016;9(4):1–13. doi:10.1161/CIRCHEARTFAILURE.115.002760.

19. Wang L, Halliday G, Huot JR, Satoh T, Baust JJ, Fisher A, et al. Treatment with Treprostinil and Metformin Normalizes Hyperglycemia and Improves Cardiac Function in Pulmonary Hypertension Associated with Heart Failure with Preserved Ejection Fraction. Arterioscler Thromb Vasc Biol. 2020;40(6):1543–1558. doi:10.1161/ATVBAHA.119.313883.

20. Miranda-Silva D, Wüst RCI, Conceição G, Gonçalves-Rodrigues P, Gonçalves N, Gonçalves A, et al. Disturbed cardiac mitochondrial and cytosolic calcium handling in a metabolic risk-related rat model of heart failure with preserved ejection fraction. Acta Physiol. 2020;228(3):1–17. doi:10.1111/apha.13378.

21. Abdellatif M, Trummer-Herbst V, Koser F, Durand S, Adão R, Vasques-Nóvoa F, et al. Nicotinamide for the treatment of heart failure with preserved ejection fraction. Sci Transl Med. 2021;13(580):eabd7064. doi:10.1126/scitranslmed.abd7064.

22. van Deel ED, Najafi A, Fontoura D, Valent E, Goebel M, Kardux K, et al. In vitro model to study the effects of matrix stiffening on Ca2+ handling and myofilament function in isolated adult rat cardiomyocytes. J Physiol. 2017;595(14):4597–4610. doi:10.1113/JP274460.

23. McCain ML, Yuan H, Pasqualini FS, Campbell PH, Parker KK. Matrix elasticity regulates the optimal cardiac myocyte shape for contractility. Am J Physiol - Hear Circ Physiol. 2014;306(11):1525–1539. doi:10.1152/ajpheart.00799.2013.

24. Hersch N, Wolters B, Dreissen G, Springer R, Kirchgessner N, Merkel R, et al. The constant beat: cardiomyocytes adapt their forces by equal contraction upon environmental stiffening. Biol Open. 2013;2(3):351–361. doi:10.1242/bio.20133830.
