# Supplementary material for: In silico identification of potential calcium dynamics and sarcomere targets for recovering left ventricular function in rat heart failure with preserved ejection fraction
Source: PLoS Comput Biol. 2021 Dec 6;17(12):e1009646. doi: 10.1371/journal.pcbi.1009646 (PMC8675924; doi:10.1371/journal.pcbi.1009646)
Supplement: S6 Text — (PDF) [file pcbi.1009646.s006.pdf]

S6 *In silico* recovering HFpEF condition towards healthy condition

In order to recover the LV function in the created ZSF1 rat HFpEF animal model, we re-parametrised the calcium dynamics or the thin filament kinetics or the thick filament kinetics or all the three components combined such that the obtained model matched the variability observed for the LV function in the healthy, control SHAM rat model.

Fig S6.1. ZSF1 and SHAM reference rat models. ZSF1 rat (red) and SHAM rat (blue) models' pressure (P) and volume (V) transients and PV-loops are plotted with dashed lines. SHAM model *in silico* variability is also represented by a cloud of thin blue full lines.

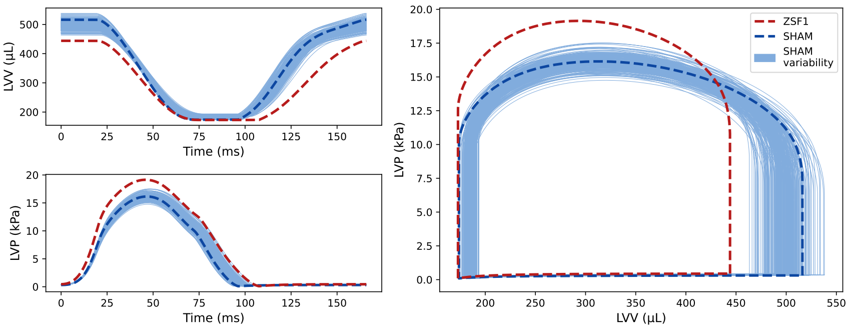

Table S6.1. SHAM rat model *in silico* variability on target LV features for ZSF1 recovery. Target mean and standard deviation LV features values to be matched for recovering the ZSF1 rat.

LV feature	Synthetic mean	Synthetic std
EDV	498.22	13.21
ESV	185.12	4.08
PeakP	16.14	0.49
maxdP	0.95	0.04
Tau	6.52	0.40

Table S6.2. GPEs' accuracy for the Ca parameter group.

LV feature	R^2	$ISE_2(\%)$
EDV	0.9984 ± 0.0009	90.4286 ± 6.7013
ESV	0.9868 ± 0.0105	98.0952 ± 3.8095
PeakP	0.9940 ± 0.0045	93.3333 ± 5.7143
maxdP	0.9965 ± 0.0008	95.2381 ± 4.2592
Tau	0.9419 ± 0.0367	90.4286 ± 6.7013

Table S6.3. GPEs' accuracy for the TNF parameter group.

LV feature	R^2	$ISE_2(\%)$
EDV	0.9725 ± 0.0182	92.2462 ± 4.8666
ESV	0.9810 ± 0.0083	93.0769 ± 5.6527
PeakP	0.9557 ± 0.0235	89.9385 ± 4.5862
maxdP	0.9766 ± 0.0203	96.8923 ± 2.8871
Tau	0.9378 ± 0.0225	86.7385 ± 11.9780

Table S6.4. GPEs' accuracy for the TKF parameter group.

LV feature	R^2	$ISE_2(\%)$
EDV	0.9802 ± 0.0159	92.1344 ± 6.3689
ESV	0.9952 ± 0.0024	92.0949 ± 7.4911
PeakP	0.9937 ± 0.0020	92.1344 ± 4.2283
maxdP	0.9588 ± 0.0296	97.3913 ± 3.4783
Tau	0.8079 ± 0.1413	90.3162 ± 7.5389

Table S6.5. GPEs' accuracy for the CaMYO parameter group.

LV feature	R^2	$ISE_2(\%)$
EDV	0.9040 ± 0.0074	91.4079 ± 4.4213
ESV	0.6910 ± 0.0214	94.7925 ± 1.8154
PeakP	0.6986 ± 0.0639	94.4755 ± 1.8533
maxdP	0.9009 ± 0.0232	92.6434 ± 3.5477
Tau	0.7994 ± 0.0515	89.5478 ± 4.7214

Table S6.6. Details of history matching progression. History matching is run for each parameter group in order to re-fit the specific parameters within the group. Parameter points ($400,000$ at Wave 1 and $100,000$ at the next Waves) are tested against an implausibility criterion using the reported cutoff values, and only a percentage of these points resulted to be non-implausible (NIMP).

**Wave**	**Parameter group**							
	**Ca**		**TNF**		**TKF**		**CaMYO**	
	cutoff	NIMP (%)	cutoff	NIMP (%)	cutoff	NIMP (%)	cutoff	NIMP (%)
1	4.5	0.08	4.0	0.01	4.0	0.07	4.0	13.75
2	4.5	0.18	4.0	0.33	4.0	0.14	4.0	73.78
3	4.2	6.53	4.0	0.80	–	–	3.5	55.03
4	4.2	92.79	–	–	–	–	3.5	84.39
5	–	–	–	–	–	–	3.0	55.98
6	–	–	–	–	–	–	3.0	84.43
7	–	–	–	–	–	–	2.5	47.94
8	–	–	–	–	–	–	2.5	83.97
9	–	–	–	–	–	–	2.0	34.12
10	–	–	–	–	–	–	2.0	75.03

Table S6.7. Percentage perturbations of cardiac cellular properties resulting from the last waves' parameter spaces. For each parameter last wave distribution, its median value is given as a ± percentage perturbation from the corresponding ZSF1 reference value.

**Group**	**Parameter**	**ZSF1 (reference)**	**RECOV (median)**	**Percentage of change**	**Direction of change**
Ca	DCA	1.2674	1.3440	6.05	↑
	AMPL	0.9977	0.8399	−15.82	↓
	TP	1.0000	1.6379	63.79	↑
	RT50	1.0000	0.6906	−30.95	↓
TNF	Ca_{50}	2.17232	2.46994	13.70	↑
	β_1	−1.50	−2.91	−93.91	↓
	k_{off}	0.0515	0.0394	−23.47	↓
	n_{trpn}	2.00	2.05	2.28	↑
TKF	k_{xb}	0.0172	0.0147	−14.18	↓
	n_{xb}	5.00	4.55	−9.07	↓
	$TRPN_{50}$	0.35	0.42	20.24	↑
	T_{ref}	156.067	222.420	42.52	↑
CaMYO	DCA	1.2674	1.4200	12.04	↑
	AMPL	0.9977	1.5486	55.22	↑
	TP	1.0000	1.4828	48.28	↑
	RT50	1.0000	0.5247	−47.53	↓
	Ca_{50}	2.1723	2.1268	−2.10	↓
	β_1	−1.50	−1.57	−4.86	↓
	k_{off}	0.0515	0.0492	−4.44	↓
	n_{trpn}	2.00	1.79	−10.69	↓
	k_{xb}	0.0172	0.0130	−24.44	↓
	n_{xb}	5.00	6.28	25.61	↑
	$TRPN_{50}$	0.35	0.48	37.12	↑
	T_{ref}	156.067	147.785	−5.31	↓
