# Supplementary material for: In silico identification of potential calcium dynamics and sarcomere targets for recovering left ventricular function in rat heart failure with preserved ejection fraction
Source: PLoS Comput Biol. 2021 Dec 6;17(12):e1009646. doi: 10.1371/journal.pcbi.1009646 (PMC8675924; doi:10.1371/journal.pcbi.1009646)

S7 Model response to input parameters' variation

We used the full simulator of the healthy rat heart contraction mechanics to investigate the EDV, ESV, SV and EF features' dependence on 5 model parameters, namely DCA, AMPL, Ca_{50}, n_{trpn}, $TRPN_{50}$, that were shown to have the highest impact on the LV features' total variance in the performed global sensitivity analysis (main manuscript, Table 3). These were further compared with the T_{ref} parameter which instead showed a limited impact on the same features. In Fig S7.1, we can see that the relationship between the considered input parameters and output features is non-linear and non-monotonic. Moreover, we can see that the parameters identified as the most important by the GSA are operating at a maximum, where a small change can cause a sharp variation in the slope of LV features' variation, whereas T_{ref} is operating on a stable slope where its impact is saturating.

Fig S7.1. Rat heart contraction model LV output features' response to model parameters' one-at-a-time variation. The full 3D biventricular rat heart contraction model is run at a fixed, reference parameter set (S5 Text, Table S5.2) with only one parameter taking equally-spaced values in the $\pm 50\,\%$ range of perturbation from its baseline value (vertical red dashed lines). The converging mechanics simulations' output PV loops are analysed to extract the corresponding EDV, ESV, SV and EF features' values (open blue dots), given as percentages from their baseline values (horizontal red dashed lines). The process is repeated separately for 6 model parameters, namely DCA, AMPL, Ca_{50}, n_{trpn}, $TRPN_{50}$, T_{ref}. A linear regression (LR) model with second-order degree polynomials is fitted to the data (blue lines) to facilitate visualisation of non-linear and non-monotonic relationships between the features and each of the parameters considered. Shaded red areas represent regions of SV feature variability when the parameters are perturbed from their baseline values, with reported percentage values being the difference between maximum and minimum SV feature perturbation.

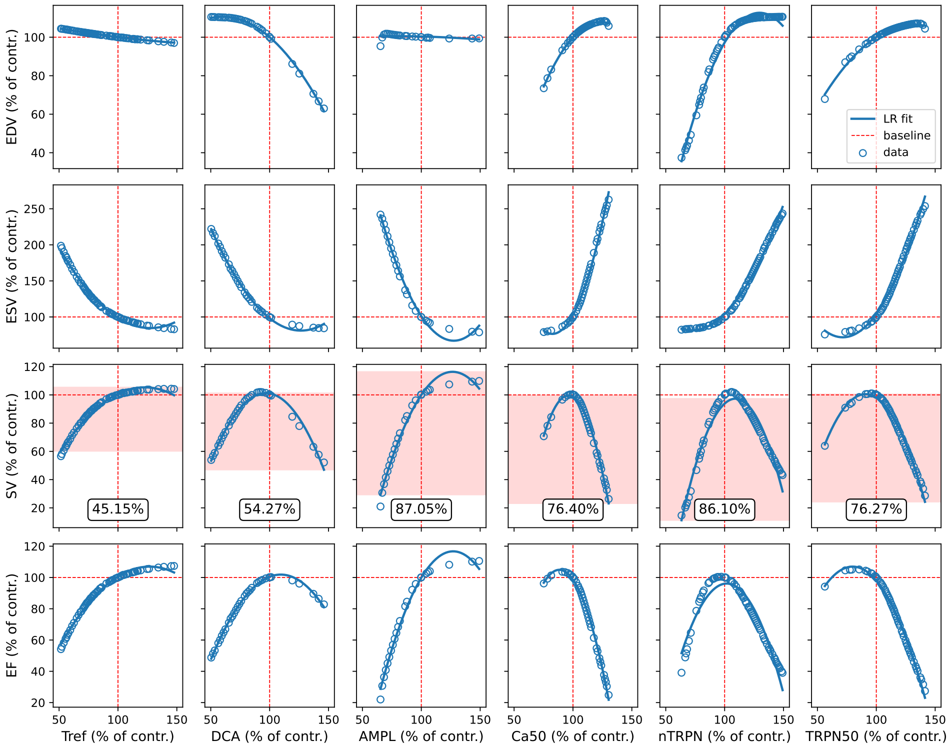

Supplement: S7 Text — (PDF) [file pcbi.1009646.s007.pdf]
